# Sequence-based approach for rapid identification of cross-clade CD8+ T-cell vaccine candidates from all high-risk HPV strains

**DOI:** 10.1007/s13205-015-0352-z

**Published:** 2016-01-27

**Authors:** Krishna P. Singh, Neeraj Verma, Bashir A. Akhoon, Vishal Bhatt, Shishir K. Gupta, Shailendra K. Gupta, Suchi Smita

**Affiliations:** 1 Department of Bioinformatics, CSIR-Indian Institute of Toxicology Research, Lucknow, 226001 India; 2 Society for Biological Research and Rural Development, Kanpur, 208014 India; 3 Department of Bioinformatics, Biocenter, University of Würzburg, Am Hubland, 97074 Würzburg, Germany; 4 Department of Systems Biology and Bioinformatics, University of Rostock, 18057 Rostock, Germany

**Keywords:** HPV, Epitope, Cytotoxic, T lymphocytes, Cervical cancer, Vaccine

## Abstract

Human papilloma virus (HPV) is the primary etiological agent responsible for cervical cancer in women. Although in total 16 high-risk HPV strains have been identified so far. Currently available commercial vaccines are designed by targeting mainly HPV16 and HPV18 viral strains as these are the most common strains associated with cervical cancer. Because of the high level of antigenic specificity of HPV capsid antigens, the currently available vaccines are not suitable to provide cross-protection from all other high-risk HPV strains. Due to increasing reports of cervical cancer cases from other HPV high-risk strains other than HPV16 and 18, it is crucial to design vaccine that generate reasonable CD8+ T-cell responses for possibly all the high-risk strains. With this aim, we have developed a computational workflow to identify conserved cross-clade CD8+ T-cell HPV vaccine candidates by considering E1, E2, E6 and E7 proteins from all the high-risk HPV strains. We have identified a set of 14 immunogenic conserved peptide fragments that are supposed to provide protection against infection from any of the high-risk HPV strains across globe.

**Electronic supplementary material:**

The online version of this article (doi:10.1007/s13205-015-0352-z) contains supplementary material, which is available to authorized users.

## Introduction

Cervical cancer is the second most common malignant cancer in terms of incidence and mortality rates in women worldwide after breast cancer (Pisani et al. 1993; Collins et al. 2006; Jemal et al. 2011; Senapathy et al. 2011; Torre et al. 2015). Human papilloma virus (HPV) is considered as the major etiological agent for cervical cancer which is responsible for over 265,700 total women deaths per year with around 527,600 new cases every year (Torre et al. 2015). It is estimated that about 80 % of women could acquire a HPV infection in their lifetime (Baseman and Koutsky 2005). On an average, every woman who dies due to cervical cancer loses about 26 years of life which is considerably greater than the average years of life lost to breast cancer (19.2 years) (Herzog and Wright 2007; Pandhi and Sonthalia 2011). Till now over 200 types of HPV strains have been sequenced (Bernard et al. 2010; Meiring et al. 2012; McLaughlin-Drubin 2015; Supindham et al. 2015), among them 16 are known to be high-risk virus strains (16, 18, 31, 33, 35, 39, 45, 51, 52, 56, 58, 59, 68, 69, 73, and 82) (Muñoz et al. 2003; Markowitz et al. 2007; Sumalee et al. 2008; Meijer et al. 2009; Dames et al. 2014) with oncogenic HPV DNAs detected in 99 % of the cervical cancers (Dunne et al. 2007). High-risk HPV strains are in most of the cases identified as main etiological agents in cervical, anal, and other genital cancers, among them about 70 % of cervical cancers worldwide are only due to HPV strains 16 and 18 (Dunne et al. 2007). For the primary prevention of cervical cancer, HPV vaccines are widely used as an important added tool (Ault et al. 2004; Fife et al. 2004; Liu et al. 2012). Prophylactic and the therapeutic formulation are the two strategies for cervical cancer vaccine candidates. Recently, FDA approved a vaccine named ‘Gardasil 9’ that protects females between the ages of 9–26 against nine HPV strains. More specifically, the first generation of Gardasil vaccine was capable to provide protections against four HPV strains (HPV-6, HPV-11, HPV-16, and HPV-18) while the latest approved version of Gardasil vaccine (Gardasil 9) provides protection against five additional high-risk HPV strains (HPV-31, HPV-33, HPV-45, HPV-52, and HPV-58) responsible for almost 20 % of cervical cancers worldwide (Braaten and Laufer 2008; Petrosky et al. 2015). Gardasil and Cervarix are prophylactic vaccines and offer no therapeutic benefit for persons already infected with HPV (Wain 2010; Pandhi and Sonthalia 2011). Most of the vaccine candidates that can be used as therapeutic vaccines include immunogenic fragments from early proteins (targets for cellular immunity) for the resolution of precancerous lesions and cervical cancer (Huh and Roden 2008). The protection offered by current vaccines is primarily against HPV types (16 and 18), although cross-protection for other high-risk HPV strains cannot be neglected. The identification of highly conserved cross-clade epitopes suitable for therapeutic intervention is clearly a crucial prerequisite for epitope-based vaccines development and for diagnostic tests to distinguish infection from high or low risk HPV strains. Cytotoxic T-cell (CTL) cross-reactivity is alleged to play an essential role in generating immune responses (Frankild et al. 2008; Petrova et al. 2012). HPV-specific cytotoxic CD8+ T lymphocytes immune responses can be detected in all untreated cervical cancer patients (Eiben et al. 2002; Valdespino et al. 2005). In animal models, cell-mediated immunity is considered to be an important mechanism for abolition of subclinical or neoplasic virus-infected cells, particularly CTL which lyses tumor cells in an antigen-specific manner (Chen et al. 1991; Feltkamp et al. 1993; De Bruijn et al. 1998; Torres-Poveda et al. 2014). Instead of individual epitopes, the use of the full-length protein with a great amount of immunogenic peptides may have a better chance to induce specific CTLs, as shown previously (Strobel et al. 2000; Adams et al. 2003; Bet et al. 2015) but the protection from all high-risk HPV strains from single vaccination will still be a question. Selection of immunogenic consensus conserved epitopes from the proteins of all HPV high-risk strains may provide an experimental basis for designing of universal HPV T-cell vaccines. In the present work, we identified conserved consensus immunogenic CD8+ T-cell epitopes from the proteome of all high-risk HPV strains and proposed a peptide pool with the ability to show immunogenic responses against all the known high-risk HPV strains.

## Materials and methods

### Sequence retrieval

Complete proteome of all the 16 high-risk HPV strains was retrieved from UniProt Knowledgebase (Apweiler et al. 2004). Amino acid sequences of four HPV proteins E1, E2, E6 and E7 were extracted from all the selected proteomes. Subsequently, four sequence datasets were manually prepared for each of the proteins from 16 high-risk HPV strains.

### Conservancy analysis

For conservancy analysis, sequence datasets were first individually aligned using ClustalW software. The substitution model was set to BLOSUM, since the mentioned substitution matrix is based on amino acid pairs in blocks of aligned protein segments, hence performs better in alignments and homology searches compared to those based on accepted mutations in closely related groups (Henikoff and Henikoff 1992). Conservancy of the amino acids of HPV strains among the aligned sequences of datasets was estimated by Shannon entropy function using protein variability server (PVS). Shannon entropy analysis (Shannon 1948) is one of the most sensitive tools to estimate the diversity of a system. For a multiple protein sequence alignment, the Shannon entropy (*H*) for every position is calculated as follows

$$H = - \mathop \sum \limits_{i = 1}^{M} {\text{P}}_{\text{i}} { \log }_{2} {\text{P}}_{\text{i}}$$

where Pi is the fraction of residues of amino acid type *i* and *M* is the number of amino acid types.

Consequently, consensus sequences were created for individual conserved fragments within Shannon entropy threshold 2 from corresponding aligned sequence datasets (Gupta et al. 2012). The consensus sequences were further used for the identification of MHC binders.

### Prediction of MHC binders

The most selective step in the presentation of antigenic peptides to T-cell receptor (TCR) is the binding of the peptide to the MHC molecule (Yewdell and Bennink 1999). NetMHC 3.2 server was used to predict binding of peptides to a number of different HLA alleles using artificial neural networks (ANNs) and weight matrices. Position specific scoring matrices (PSSMs)-based SYFPEITHI (Rammensee et al. 1999) method was employed for the prediction of binding peptides of major histocompatibility complex (MHC) class I. NetMHC 3.2 predicts epitopes with length ranging from 8 to 11 amino acids. Only epitopes with IC50 value below 500 nM were considered as the potential epitopes in ANNs-based prediction from the consensus fragments for four protein datasets. As the epitopes were predicted from the consensus sequences of conserved protein fragments, these epitopes were again realigned with E1, E2, E6 and E7 proteins of all high-risk HPV strains. Only those epitopes were kept for subsequent analyses which were 100 % identical to their respective proteins. Further, we performed common allele matrix analysis (Gupta et al. 2010) to discard those epitopes whose allelic response is coverd by more efficient epitopes from the same high-risk HPV strain.

### Prediction of non-human homologous immunogenic peptide fragments

All the overlapping epitopes (9 mer) were merged to design the potential immunogenic peptide fragments for CD8+ response from each of the protein datasets. A number of immunogenic peptides may fail to raise a T-cell response if they are recognized as self peptides due to significant similarity exists between the antigenic peptides and proteins of host organism. For this, the final sets of potential immunogenic peptide fragments were checked for their similarities with annotated human proteins using offline BLAST similarity search.

### Population coverage analysis

Population coverage analysis plays a significant role in the epitope-based vaccine designing because of the highly polymorphic nature of the MHC molecules (Reche and Reinherz 2003; Stern and Wiley 1994). Population coverage of newly identified immunogenic peptide fragments were individually analyzed using population coverage tool available at IEDB web server. The tool calculates the response of individuals set of peptide fragments with known MHC- I restrictions on the basis of maximum HLA binding alleles. The average projected population coverage of class I epitopes for the populations distributed in various human populations (ethnicities) was estimated.

## Results

The E1, E2, E6 and E7 protein sequences from all high-risk HPV strains were collected from UniProtKB and aligned using multiple sequence alignment software clustalW. The multiple alignment files for all the four protein data sets with conserved regions are shown in Supplementary File 1. The conserved fragments within aligned strains of sequence datasets were analyzed by PVS with variability threshold (*H*) ≤ 2.0. Typically, positions with *H* ≥ 2.0 are considered variable, whereas those with *H* ≤ 1 are consider highly conserved (Litwin and Jores 1992). Additionally the positions with *H* ≤ 2 and *H* > 1 are also considered as conserved regions (Gupta et al. 2009, 2011). The default cutoff value (*H* = 1) was too strict, resulting in fewer and smaller targets for the following analysis so the cutoff threshold value was set as 2.0 in the present analysis to identify reasonable conserved fragments with length ≥9 amino acid residues. The consensus sequences of all the conserved regions with length ≥9 amino acids in four datasets of E1, E2, E6 and E7 proteins from high-risk HPV strains are shown in Table 1.

**Table 1:** Conserved protein fragments from E1, E2, E6 and E7 protein datasets from all high-risk HPV strains

HPV protein	Conserved fragment number	Start position	End position	Sequence with nine or more consecutive conserved residues filtered from protein variability server
E1	E1-1	2	12	MADPEGTDGEG
	E1-2	21	31	VEAIVEKKTGD
	E1-3	33	41	ISDDEDENA
	E1-4	66	76	ETAQALFNAQE
	E1-5	135	146	PDSGYGNTEVET
	E1-6	241	257	ELVRPFKSDKTTCTDWV
	E1-7	265	278	PSVAEGLKTLIKPY
	E1-8	291	307	WGVIILMLIRFKCGKNR
	E1-9	312	321	KLLSTLLNVP
	E1-10	324	334	CMLIEPPKLRS
	E1-11	336	352	AAALYWYRTGISNISEV
	E1-12	362	373	RQTVLQHSFDDS
	E1-13	375	388	FDLSEMVQWAFDND
	E1-14	406	447	NSNAAAFLKSNCQAKYVKDCATMCRHYKRAQKRQMSMSQWIK
	E1-15	455	474	DGGDWRPIVQFLRYQGVEFI
	E1-16	483	516	FLKGTPKKNCIVIYGPANTGKSYFGMSLIHFLQG
	E1-17	535	545	DAKIAMLDDAT
	E1-18	555	572	YMRNALDGNPISIDRKHR
	E1-19	574	590	LVQLKCPPLLITSNINP
	E1-20	595	606	RWPYLHSRLTVF
	E1-21	624	642	INDKNWKSFFSRTWSRLDL
	E1-22	645	653	EEEDKENDG
E2	E2-1	7	26	METLSQRLNVCQDKILDHYE
	E2-2	45	54	ECAIFYKARE
	E2-3	77	89	QAIELQMALESLN
	E2-4	110	118	TEPKKCFKK
	E2-5	204	212	CPESVSSTS
	E2-6	305	314	TTPIVHLKGD
E6	E6-1	14	23	ERPRKLHDLC
	E6-2	25	33	ALETSLHDI
	E6-3	76	86	FYSKISEYRHY
	E6-4	113	127	CQKPLCPEEKQRHLD
	E6-5	129	137	KKRFHNIAG
E7	E7-1	6	15	PTLQDIVLDL
	E7-2	95	103	LQQLLMGTL

To generate immunological responses against any pathogen, the binding of antigenic peptide from pathogen and its binding affinity to MHC molecules is one of the crucial steps (Roomp et al. 2010). Using NetMHC 3.2 server, we predicted a total of 65 unique epitopes from the consensus sequences dataset of E1 protein. Likewise, 8 from E2, 5 from E6 and 2 unique epitopes from E7 protein consensus sequence datasets were predicted by the server with either strong or weak binding affinity (Supplementary Table 1–4). As epitopes were predicted from the consensus conserved peptide fragments of protein datasets through NetMHC 3.2 server, there are chances that some of the epitopes may not show 100 % identity with any of the protein in the datasets due to presence of consensus amino acid residues substituted during conservancy analysis. To verify this, epitopes were realigned with the primary protein datasets of E1, E2, E6 and E7 proteins collected for various high-risk HPV strains. Those epitopes which were not completely mapped with any of the primary protein sequences of high-risk HPV strains were filtered out. In this process, 16 epitopes from E1, 2 from E2, 2 from E6 and both the epitopes from E7 datasets were discarded. To predict the long immunogenic peptide fragments, all the overlapping epitopes were merged (Table 2). In total, 15 immunogenic conserved peptide fragments were identified, of which 11 fragments were from E1, 3 from E2 and 1 fragment was from E6 protein dataset of high-risk HPV strains.

**Table 2:** Immunogenic peptide fragments identified from selected protein datasets of high-risk HPV strains

HPV protein fragment		Immunogenic peptide sequence	HLA alleles targeted	High-risk HPV strains mapped
Protein name	Immunogenic peptide fragment			
E1	E1-F1	DSGYGNTEV	HLA-A6802	16, 31, 33, 58, 73
	E1-F2	LVRPFKSDK	HLA-A0301,HLA-A3001	33, 58
	E1-F3	CMLIEPPKL	HLA-A0201,HLA-A0211,HLA-A0212,HLA-A0216,HLA-A0219,HLA-A0250	45, 59
	E1-F4	ALYWYRTGISNISEV	HLA-A0201,HLA-A0202,HLA-A0203,HLA-A0206,HLA-A0211,HLA-A0212,HLA-A0216,HLA-A0250,HLA-A2301,HLA-A2403,HLA-A6802	16, 18, 39, 45, 68
	E1-F5	DLSEMVQWAFD	HLA-A0203,HLA-A0211,HLA-A0216,HLA-A0219,HLA-A0250,HLA-B4501	18, 33, 35
	E1-F6	NSNAAAFLKSNCQAKYVKDCATMCRHYKRAQKRQMSMSQWIK	HLA-A0201, HLA-A0202,HLA-A0203,HLA-A0206,HLA-A2402,HLA-A3201,HLA-B1501,HLA-B1503,HLA-B2705,HLA-A0301,HLA-A1101,HLA-A6801,HLA-B1503,HLA-A3001,HLA-B0702,HLA-B0801,HLA-B1517,HLA-B5801,HLA-A3101,HLA-A3301,HLA-A2301,HLA-A2403,HLA-A3002,HLA-A6801	39, 59, 16, 68, 45, 31, 35, 52, 69, 39, 51, 82, 18
	E1-F7	WRPIVQFLRYQGVEFI	HLA-B2705,HLA-B3501, HLA-B5301,HLA-A0203,HLA-A0206,HLA-A0211,HLA-A0216,HLA-A0250, HLA-B1503	18, 39, 45, 58, 68, 56, 59
	E1-F8	PKKNCIVIYGPANTGKSYFGMSLIHFL	HLA-A0201,HLA-A0202,HLA-A0206,HLA-A0211,HLA-A0216,HLA-A6802,HLA-A6901,HLA-B3901,HLA-A2301,HLA-A2402, HLA-A2403,HLA-A2902,HLA-B1501,HLA-A0203,HLA-A3001,HLA-A3201,HLA-B1517,HLA-B5801,HLA-B1503,HLA-A2603,HLA-B1502,HLA-B3501,HLA-B1503	18, 58, 31, 33, 52, 59, 45, 39
	E1-F9	VQLKCPPLLITSNI	HLA-B5301,HLA-A0203,HLA-A0201,HLA-A0206,HLA-B1503,HLA-B3901,HLA-B4801	16, 31, 33, 35, 51
	E1-F10	RWPYLHSRLTVF	HLA-A0202,HLA-A0203,HLA-A0211,HLA-A0250,HLA-B0801,HLA-B1501,HLA-B1502,HLA-B1503,HLA-B1517,HLA-B3501,HLA-B5401,HLA-A2402,HLA-A2403	31, 33, 52, 58, 68
	E1-F11	KNWKSFFSRTWSRL	HLA-A2301,HLA-A2403,HLA-A1101,HLA-A3101,HLA-A3301,HLA-A6801,HLA-B1503,HLA-A3101	16, 31, 33, 35, 52, 58
E2	E2-F1	ETLSQRLNVCQDKI	HLA-A0202,HLA-A0203,HLA-A0212,HLA-A0219,HLA-A6802,HLA-A6901	16, 31
	E2-F2	ECAIFYKAR	HLA-A6801	69
	E2-F3	QAIELQMALESL	HLA-A0202,HLA-A0211,HLA-A0219,HLA-A0250,HLA-B4002,HLA-A0206,HLA-A6802,HLA-A6901,HLA-B3501,HLA-B3901	39, 59, 68
E6	E6-F1	FYSKISEYRHY	HLA-A2602,HLA-B1503,HLA-B1517,HLA-A2403,HLA-A3101,HLA-A3301,HLA-A6801	16, 33, 35, 52, 58

**Fig. 1:**
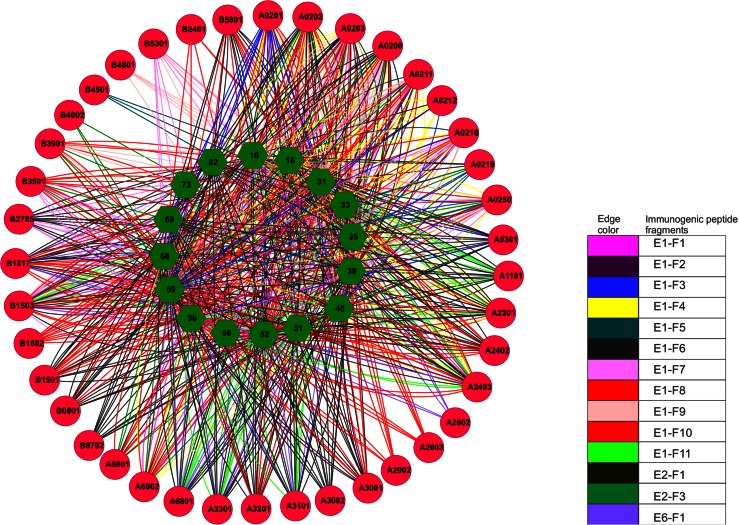
Illustration of the interaction network of immunogenic peptide fragments, High-risk HPV strains which contributed for the formation of these fragments and MHC class I alleles are shown. Edges with* different colors* represent various immunogenic peptide fragments as shown in figure legends. High-risk HPV strains are shown in* green hexagon* in the* inner circle* and HLA alleles on the* outer circle* with* red color*. From the figure it is clear that few alleles (e.g. A2602, A2603, A2902, A3002, B0702, B1502, B4002, B4501, B4801, B5401) are targeted by only one immunogenic peptide fragments, while majority of HLA alleles (A0201, A0202, A0203, A0206, A0211, A0212, A0216, A0219, A0250, A0301, A1101, A2301, A2402, A2403, A3001, A3101, A3201, A3301, A6801, A6802, A6901, B0801, B1501, B1503, B1517, B2705, B3501, B3901, B5301, B5801) are targeted by most of the immunogenic peptides selected in this study

To further identify the minimal set of immunogenic peptide fragment datasets to target all the high-risk HPV strains, we discarded those peptide fragments where the targeted HLA alleles and high-risk HPV strains were the subsets of other highly efficient peptide fragments shown in Table 2. In this process, immunogenic peptide sequence ECAIFYKAR (Table 2: E2-F2) from the high-risk HPV strain 69 was filtered out as it can be considered the subset of immunogenic fragment E1-F6 which is also present in type 69 HPV strain and also target the same allele besides its affinity with other alleles. Thus, in total, we have identified a peptide pool of 14 sequences that might be effective to generate immunogenic responses against any of the high-risk HPV strains. The interaction map of immunogenic peptide fragments, their HLA allelic responses and their presence in various HPV high-risk strains are shown in Fig. 1. To discard peptide fragments that can be recognized as self protein for the immune system we performed BLAST screening of all the 14 immunogenic peptides with entire human proteome. None of the peptide was similar to human proteome. We finally performed the population coverage analysis of generated immunogenic peptide pool using population coverage analysis tool available at IEDB server (http://tools.immuneepitope.org/tools/population). The percentage population coverage of the immunogenic peptide pool generated from the current analysis in various ethnicities is shown in Table 3.

**Table 3:** Population coverage analysis for immunogenic peptide pooled in various ethnicities

High-risk HPV strain	Percentage population coverage in various ethnicities									
	Australia (%)	Europe (%)	North Africa (%)	North America (%)	North-East Asia (%)	Oceania (%)	South America (%)	South-East Asia (%)	South-West Asia (%)	Sub-Saharan Africa (%)
TYPE 16	81.24	94.35	63.21	95.17	69.98	88.73	53.15	86.72	79.18	85.14
TYPE 18	65.40	74.28	50.63	91.46	60.15	76.33	53.56	73.07	64.57	76.17
TYPE 31	81.33	95.31	65.99	96.89	76.13	91.12	54.37	89.97	81.73	86.83
TYPE 33	80.85	92.30	64.35	95.03	74.60	91.06	53.76	89.76	80.19	83.36
TYPE 35	81.24	94.15	62.30	95.14	69.90	88.73	52.88	86.69	78.19	82.91
TYPE 39	81.29	95.30	65.99	95.88	71.40	89.45	54.17	87.99	81.60	86.83
TYPE 45	81.29	95.30	65.99	95.88	71.40	89.45	54.17	87.99	81.60	86.83
TYPE 51	81.24	94.04	56.96	95.04	68.92	88.73	48.71	86.55	77.50	79.99
TYPE 52	81.29	95.30	65.99	95.88	74.42	89.58	54.17	88.72	81.51	86.83
TYPE 56	0.49	17.12	8.63	40.71	18.50	2.56	8.86	14.30	15.98	23.50
TYPE 58	80.85	92.75	59.07	95.17	74.34	89.51	53.56	88.50	79.96	80.90
TYPE 59	81.29	95.30	65.99	95.88	71.40	89.45	54.17	87.99	81.60	86.83
TYPE 68	81.29	95.30	65.99	95.88	73.51	89.58	54.17	88.47	81.60	86.83
TYPE 69	80.40	93.85	55.85	93.45	65.25	86.26	44.60	83.12	77.29	79.75
TYPE 73	0.00	1.58	9.82	0.92	0.00	0.00	0.00	0.03	1.63	14.68
TYPE 82	80.40	93.85	55.85	93.45	65.25	86.26	44.60	83.12	77.29	79.75

## Discussion

In the post-genomic era, strategies of vaccine development have progressed dramatically from traditional Pasteur’s principles of isolating, inactivating and injecting the causative agent of an infectious disease, to reverse vaccinology that initiates from in silico analysis of the genome information (Akhoon et al. 2011). In silico works have attracted considerable attention of experimental biologists for rapid screening and identification of probable vaccine candidates (Sakib et al. 2014; Sharma et al. 2013; Hasan et al. 2013; Oany et al. 2014). The availability of fully sequenced proteome from high-risk HPV strains provide an opportunity for computer-aided screening of reliable peptide-based therapeutic vaccines candidates among billions of possible immune-active peptides.

We extracted high-risk HPV proteome for 8 HPV specific proteins designated as E- (E1, E2, E4, E5, E6 and E7) or L-type (L1 and L2) according to their expression in early or late differentiation stage of the epithelium (Burd 2003; Rautava and Syrjänen 2012). The early secretary proteins expressed during early differentiating stage of epithelium development in all the 16 high-risk HPV strains. Since the E5 protein of HPV has previously been reported as inducer of down-regulation of MHC class I, we ignored E5 protein in the current analysis. Moreover, E2–E5 region has been shown to be lost when the episomal HPV DNA integrates into host chromosome; using E2–E5 proteins as vaccine candidates may be futile. However, since E1 and E2 are expressed in higher levels than E6 and E7 early in the progress of an HPV infection, it may be assumed that these proteins may considered as good targets for vaccine designing to treat early stages of disease (Burd 2003). Therefore, E2 protein was included for epitope-based vaccine designing in the present study along with E1, E6 and E7 protein. Based on our computational workflow, we generated a pool of 14 peptide fragments with length varying from 9 to 43 amino acid residues to provide immunogenic responses against all the high-risk HPV strains. The hallmark of the immune system is its ability to recognize and distinguish between self and non-self. T-cells do this task by recognizing peptides that are bound to MHC receptors. Epitopes are usually thought to be derived from non-self protein antigen that interacts with antibodies or T-cell receptors, thereby activating an immune response. Epitopes are usually thought to be derived from non-self protein antigen that interacts with antibodies or T-cell receptors, thereby activating an immune response. Also the antigen–antibody interaction is reversible, therefore, weak interactions often lead to cross-reactivity of antigens. The main reasons of cross-reactivity are either the homologous proteins that are conserved throughout the development and expressed by both the infectious agent and the host or the viral proteins that share short regions of amino acid sequence similarity with a non-homologous host protein. Therefore, to exclude the epitopes those are conserved between the pathogen and the host, we performed sequence alignment of the selected pool of 14 immunogenic peptides with entire human proteome. We did not observe similarity between any of the selected immunogenic peptide fragments with human proteome, we also discarded epitopes showing weak interactions with HLA alleles. Thus we believe that all the 14 sequences together will form the best peptide pool to generated immunogenic responses against any of the high-risk HPV strains infection.

Population coverage analysis for a given set of immunogenic peptides is important to determine their efficacy as the frequency of expression of their targeted HLA alleles varies across ethnicities. With the population coverage analysis, we concluded that least immunogenic response is shown by HPV strain 73 followed by strain 56 in almost all the ethnicities. Thus, the data indicate that the generated peptide fragments pool will provide very weak protection against HPV Type 73 and 56. The best response is shown by North American population for all the HPV high-risk strains based on the HLA alleles frequency data. Overall, we proposed the pool of 14 immunogenic peptide fragments with length ranging from 9 to 43 amino acid residues to provide the protection against all the HPV high-risk strains except Type 73 and 56 in all the ethnicities.

## Conclusion

Immunoinformatics has changed the paradigm of ancient vaccinology since it is recently emerged as a critical field for accelerating immunology research (Baloria et al. 2012). Moreover, the immunoinformatics techniques applied to T-cells have advanced to a greater degree than those dealing with B-cells. Indeed, it is now a common practice to identify the vaccine candidates using in silico approaches before being subjected to in vitro confirmatory studies (Gupta et al. 2009; Ranjbar et al. 2015). The major objective of our study was to identify an immunogenic peptide pool containing epitopes that can be effective against all the high-risk HPV strains circulating globally. Some cross-protections were already observed in case of previously identified vaccines. Such as, Cervarix vaccine which was initially designed by targeting HPV 16 and 18 strains but later found to provide additional cross-protections against HPV 31, 33 and 45 strains. However, most of the high-risk HPV strains were evolved by accumulating random mutations in the epitopes recognizing regions and therefore many of the high-risk strains are not effectively targeted by available HPV vaccines. Therefore, we used the consensus epitopes extracted from highly conserved regions of E1, E2, E6 and E7 proteins from all the high-risk HPV strains identified so far. The vaccine formulated by the proposed peptide pool can alert the body’s immune system to generate immunization memory cells upon injecting. Subsequently, as these are MHC class I epitopes, the proteasomal degradation machineries can degrade the whole vaccine and release these conserved epitopes in host. Furthermore, because of the conserved nature of the epitopes the immune system will be trained to recognize these epitopes in case of HPV infection by any high-risk strains and thus can provide the cross-protection. Using the computational workflow presented in this manuscript, we identified 14 conserved immunogenic peptide fragments from 4 early proteins (E1, E2, E6 and E7) of 16 high-risk HPV types providing CD8+ responses which can be validated experimentally for the designing of an universal vaccine against all the high-risk HPV strains.

## Electronic supplementary material

Below is the link to the electronic supplementary material.

Supplementary material 1 (DOCX 36 kb)

Supplementary material 2 (DOCX 16 kb)

Supplementary material 3 (DOCX 15 kb)

Supplementary material 4 (DOCX 14 kb)

Supplementary material 5 (DOCX 35 kb)

## Abbreviations

HPV: Human papilloma virus
CTL: Cytotoxic T-cell
PVS: Protein variability server
MHC: Major histocompatibility complex
TCR: T-cell receptor
HLA: Human leukocyte antigen
PSSMs: Position specific scoring matrices
ANN: Artificial neural network
