# Supplementary material for: Sequence-based approach for rapid identification of cross-clade CD8+ T-cell vaccine candidates from all high-risk HPV strains
Source: 3 Biotech. 2016 Jan 27;6(1):39. doi: 10.1007/s13205-015-0352-z (PMC4729761; doi:10.1007/s13205-015-0352-z)
Supplement: Supplementary file 1 — Supplementary material 1 (DOCX 36 kb) [file 13205_2015_352_MOESM1_ESM.docx]

**Supplementary Table 1:** Prediction of 9mer epitopes and their targeted alleles from the conserved consensus E1 protein fragment datasets of high-risk HPV strains

| Number of unique epitopes | Conserved fragment number* | Start position of epitope in the fragment | Epitope sequence | Affinity(nM) | Binding Level** | HLA-allele targeted |
| --- | --- | --- | --- | --- | --- | --- |
| 1 | E1-4 | 1 | ETAQALFNA | 207 | WB | HLA-A2602 |
|  | E1-4 | 1 | ETAQALFNA | 7 | SB | HLA-A6802 |
|  | E1-4 | 1 | ETAQALFNA | 6 | SB | HLA-A6901 |
| 2 | E1-5 | 2 | DSGYGNTEV | 378 | WB | HLA-A6802 |
| 3 | E1-6 | 2 | LVRPFKSDK | 257 | WB | HLA-A0301 |
|  | E1-6 | 2 | LVRPFKSDK | 8 | SB | HLA-A3001 |
| 4 | E1-7 | 2 | SVAEGLKTL | 377 | WB | HLA-A0202 |
|  | E1-7 | 2 | SVAEGLKTL | 53 | WB | HLA-A0203 |
|  | E1-7 | 2 | SVAEGLKTL | 30 | SB | HLA-A0206 |
|  | E1-7 | 2 | SVAEGLKTL | 175 | WB | HLA-A0211 |
|  | E1-7 | 2 | SVAEGLKTL | 217 | WB | HLA-A0216 |
|  | E1-7 | 2 | SVAEGLKTL | 41 | SB | HLA-A0250 |
|  | E1-7 | 2 | SVAEGLKTL | 80 | WB | HLA-A2602 |
|  | E1-7 | 2 | SVAEGLKTL | 249 | WB | HLA-A6901 |
|  | E1-7 | 2 | SVAEGLKTL | 496 | WB | HLA-B1517 |
| 5 | E1-7 | 6 | GLKTLIKPY | 90 | WB | HLA-B1501 |
|  | E1-7 | 6 | GLKTLIKPY | 451 | WB | HLA-B1502 |
|  | E1-7 | 6 | GLKTLIKPY | 450 | WB | HLA-B1503 |
| 6 | E1-8 | 2 | GVIILMLIR | 85 | WB | HLA-A1101 |
|  | E1-8 | 2 | GVIILMLIR | 213 | WB | HLA-A3101 |
|  | E1-8 | 2 | GVIILMLIR | 434 | WB | HLA-A6801 |
| 7 | E1-8 | 3 | VIILMLIRF | 58 | WB | HLA-A2602 |
|  | E1-8 | 3 | VIILMLIRF | 215 | WB | HLA-B1517 |
| 8 | E1-8 | 4 | IILMLIRFK | 69 | WB | HLA-A0301 |
|  | E1-8 | 4 | IILMLIRFK | 32 | SB | HLA-A1101 |
|  | E1-8 | 4 | IILMLIRFK | 78 | WB | HLA-A3001 |
|  | E1-8 | 4 | IILMLIRFK | 227 | WB | HLA-A3101 |
|  | E1-8 | 4 | IILMLIRFK | 463 | WB | HLA-A6801 |
| 9 | E1-8 | 5 | ILMLIRFKC | 459 | WB | HLA-A0219 |
|  | E1-8 | 5 | ILMLIRFKC | 105 | WB | HLA-A0250 |
| 10 | E1-8 | 6 | LMLIRFKCG | 69 | WB | HLA-B0801 |
| 11 | E1-8 | 7 | MLIRFKCGK | 226 | WB | HLA-A0250 |
|  | E1-8 | 7 | MLIRFKCGK | 86 | WB | HLA-A0301 |
|  | E1-8 | 7 | MLIRFKCGK | 39 | SB | HLA-A1101 |
|  | E1-8 | 7 | MLIRFKCGK | 244 | WB | HLA-A3001 |
|  | E1-8 | 7 | MLIRFKCGK | 192 | WB | HLA-A3101 |
|  | E1-8 | 7 | MLIRFKCGK | 38 | SB | HLA-A6801 |
| 12 | E1-8 | 9 | IRFKCGKNR | 153 | WB | HLA-B1503 |
|  | E1-8 | 9 | IRFKCGKNR | 216 | WB | HLA-B2705 |
| 13 | E1-9 | 1 | KLLSTLLNV | 8 | SB | HLA-A0201 |
|  | E1-9 | 1 | KLLSTLLNV | 32 | SB | HLA-A0202 |
|  | E1-9 | 1 | KLLSTLLNV | 10 | SB | HLA-A0203 |
|  | E1-9 | 1 | KLLSTLLNV | 6 | SB | HLA-A0206 |
|  | E1-9 | 1 | KLLSTLLNV | 2 | SB | HLA-A0211 |
|  | E1-9 | 1 | KLLSTLLNV | 3 | SB | HLA-A0212 |
|  | E1-9 | 1 | KLLSTLLNV | 4 | SB | HLA-A0216 |
|  | E1-9 | 1 | KLLSTLLNV | 2 | SB | HLA-A0219 |
|  | E1-9 | 1 | KLLSTLLNV | 2 | SB | HLA-A0250 |
|  | E1-9 | 1 | KLLSTLLNV | 206 | WB | HLA-A3001 |
|  | E1-9 | 1 | KLLSTLLNV | 473 | WB | HLA-A3201 |
|  | E1-9 | 1 | KLLSTLLNV | 335 | WB | HLA-A6901 |
|  | E1-9 | 1 | KLLSTLLNV | 265 | WB | HLA-B1503 |
| 14 | E1-9 | 2 | LLSTLLNVP | 484 | WB | HLA-A0202 |
| 15 | E1-10 | 1 | CMLIEPPKL | 401 | WB | HLA-A0201 |
|  | E1-10 | 1 | CMLIEPPKL | 32 | SB | HLA-A0211 |
|  | E1-10 | 1 | CMLIEPPKL | 278 | WB | HLA-A0212 |
|  | E1-10 | 1 | CMLIEPPKL | 84 | WB | HLA-A0216 |
|  | E1-10 | 1 | CMLIEPPKL | 72 | WB | HLA-A0219 |
|  | E1-10 | 1 | CMLIEPPKL | 66 | WB | HLA-A0250 |
| 16 | E1-10 | 2 | MLIEPPKLR | 262 | WB | HLA-A3101 |
|  | E1-10 | 2 | MLIEPPKLR | 261 | WB | HLA-A3301 |
|  | E1-10 | 2 | MLIEPPKLR | 56 | WB | HLA-A6801 |
| 17 | E1-11 | 3 | ALYWYRTGI | 28 | SB | HLA-A0201 |
|  | E1-11 | 3 | ALYWYRTGI | 22 | SB | HLA-A0202 |
|  | E1-11 | 3 | ALYWYRTGI | 6 | SB | HLA-A0203 |
|  | E1-11 | 3 | ALYWYRTGI | 120 | WB | HLA-A0206 |
|  | E1-11 | 3 | ALYWYRTGI | 3 | SB | HLA-A0211 |
|  | E1-11 | 3 | ALYWYRTGI | 331 | WB | HLA-A0212 |
|  | E1-11 | 3 | ALYWYRTGI | 16 | SB | HLA-A0216 |
|  | E1-11 | 3 | ALYWYRTGI | 3 | SB | HLA-A0250 |
|  | E1-11 | 3 | ALYWYRTGI | 72 | WB | HLA-A3201 |
| 18 | E1-11 | 6 | WYRTGISNI | 179 | WB | HLA-A2301 |
|  | E1-11 | 6 | WYRTGISNI | 20 | SB | HLA-A2403 |
| 19 | E1-11 | 9 | TGISNISEV | 70 | WB | HLA-A6802 |
| 20 | E1-12 | 1 | RQTVLQHSF | 185 | WB | HLA-A3201 |
|  | E1-12 | 1 | RQTVLQHSF | 115 | WB | HLA-B1501 |
|  | E1-12 | 1 | RQTVLQHSF | 8 | SB | HLA-B1503 |
|  | E1-12 | 1 | RQTVLQHSF | 106 | WB | HLA-B2705 |
|  | E1-12 | 1 | RQTVLQHSF | 104 | WB | HLA-B5801 |
| 21 | E1-13 | 1 | FDLSEMVQW | 453 | WB | HLA-B5301 |
| 22 | E1-13 | 2 | DLSEMVQWA | 274 | WB | HLA-A0203 |
|  | E1-13 | 2 | DLSEMVQWA | 395 | WB | HLA-A0211 |
|  | E1-13 | 2 | DLSEMVQWA | 334 | WB | HLA-A0216 |
|  | E1-13 | 2 | DLSEMVQWA | 256 | WB | HLA-A0219 |
|  | E1-13 | 2 | DLSEMVQWA | 21 | SB | HLA-A0250 |
| 23 | E1-13 | 3 | LSEMVQWAF | 91 | WB | HLA-A0101 |
|  | E1-13 | 3 | LSEMVQWAF | 66 | WB | HLA-B1517 |
|  | E1-13 | 3 | LSEMVQWAF | 326 | WB | HLA-B3501 |
|  | E1-13 | 3 | LSEMVQWAF | 89 | WB | HLA-B5801 |
| 24 | E1-13 | 4 | SEMVQWAFD | 438 | WB | HLA-B4501 |
| 25 | E1-14 | 1 | NSNAAAFLK | 208 | WB | HLA-A0301 |
|  | E1-14 | 1 | NSNAAAFLK | 14 | SB | HLA-A1101 |
|  | E1-14 | 1 | NSNAAAFLK | 12 | SB | HLA-A6801 |
| 26 | E1-14 | 8 | LKSNCQAKY | 46 | SB | HLA-A3002 |
|  | E1-14 | 8 | LKSNCQAKY | 23 | SB | HLA-B1503 |
| 27 | E1-14 | 9 | KSNCQAKYV | 123 | WB | HLA-A3001 |
|  | E1-14 | 9 | KSNCQAKYV | 244 | WB | HLA-B1517 |
| 28 | E1-14 | 15 | KYVKDCATM | 318 | WB | HLA-A2301 |
|  | E1-14 | 15 | KYVKDCATM | 414 | WB | HLA-A2402 |
|  | E1-14 | 15 | KYVKDCATM | 195 | WB | HLA-A2403 |
| 29 | E1-14 | 20 | CATMCRHYK | 51 | WB | HLA-A1101 |
|  | E1-14 | 20 | CATMCRHYK | 79 | WB | HLA-A3101 |
|  | E1-14 | 20 | CATMCRHYK | 256 | WB | HLA-A3301 |
|  | E1-14 | 20 | CATMCRHYK | 20 | SB | HLA-A6801 |
| 30 | E1-14 | 21 | ATMCRHYKR | 17 | SB | HLA-A1101 |
|  | E1-14 | 21 | ATMCRHYKR | 6 | SB | HLA-A3101 |
|  | E1-14 | 21 | ATMCRHYKR | 58 | WB | HLA-A3301 |
|  | E1-14 | 21 | ATMCRHYKR | 23 | SB | HLA-A6801 |
| 31 | E1-14 | 22 | TMCRHYKRA | 472 | WB | HLA-A0203 |
| 32 | E1-14 | 24 | CRHYKRAQK | 302 | WB | HLA-B2705 |
| 33 | E1-14 | 26 | HYKRAQKRQ | 113 | WB | HLA-A3001 |
| 34 | E1-14 | 27 | YKRAQKRQM | 43 | SB | HLA-B1503 |
| 35 | E1-14 | 29 | RAQKRQMSM | 153 | WB | HLA-A3001 |
|  | E1-14 | 29 | RAQKRQMSM | 95 | WB | HLA-B0702 |
|  | E1-14 | 29 | RAQKRQMSM | 68 | WB | HLA-B0801 |
|  | E1-14 | 29 | RAQKRQMSM | 98 | WB | HLA-B1503 |
|  | E1-14 | 29 | RAQKRQMSM | 57 | WB | HLA-B1517 |
|  | E1-14 | 29 | RAQKRQMSM | 132 | WB | HLA-B5801 |
| 36 | E1-14 | 32 | KRQMSMSQW | 344 | WB | HLA-B1503 |
| 37 | E1-14 | 33 | RQMSMSQWI | 129 | WB | HLA-A0201 |
|  | E1-14 | 33 | RQMSMSQWI | 144 | WB | HLA-A0202 |
|  | E1-14 | 33 | RQMSMSQWI | 76 | WB | HLA-A0203 |
|  | E1-14 | 33 | RQMSMSQWI | 17 | SB | HLA-A0206 |
|  | E1-14 | 33 | RQMSMSQWI | 495 | WB | HLA-A2402 |
|  | E1-14 | 33 | RQMSMSQWI | 59 | WB | HLA-A3201 |
|  | E1-14 | 33 | RQMSMSQWI | 234 | WB | HLA-B1501 |
|  | E1-14 | 33 | RQMSMSQWI | 18 | SB | HLA-B1503 |
|  | E1-14 | 33 | RQMSMSQWI | 376 | WB | HLA-B2705 |
| 38 | E1-14 | 34 | QMSMSQWIK | 154 | WB | HLA-A0301 |
|  | E1-14 | 34 | QMSMSQWIK | 30 | SB | HLA-A1101 |
|  | E1-14 | 34 | QMSMSQWIK | 330 | WB | HLA-A6801 |
| 39 | E1-15 | 5 | WRPIVQFLR | 237 | WB | HLA-B2705 |
| 40 | E1-15 | 6 | RPIVQFLRY | 161 | WB | HLA-A2902 |
|  | E1-15 | 6 | RPIVQFLRY | 26 | SB | HLA-A8001 |
|  | E1-15 | 6 | RPIVQFLRY | 69 | WB | HLA-B3501 |
|  | E1-15 | 6 | RPIVQFLRY | 95 | WB | HLA-B5301 |
| 41 | E1-15 | 9 | VQFLRYQGV | 61 | WB | HLA-A0203 |
|  | E1-15 | 9 | VQFLRYQGV | 12 | SB | HLA-A0206 |
|  | E1-15 | 9 | VQFLRYQGV | 80 | WB | HLA-A0211 |
|  | E1-15 | 9 | VQFLRYQGV | 282 | WB | HLA-A0216 |
|  | E1-15 | 9 | VQFLRYQGV | 417 | WB | HLA-A0250 |
|  | E1-15 | 9 | VQFLRYQGV | 94 | WB | HLA-B1503 |
| 42 | E1-15 | 11 | FLRYQGVEF | 40 | SB | HLA-B1501 |
|  | E1-15 | 11 | FLRYQGVEF | 31 | SB | HLA-B1502 |
|  | E1-15 | 11 | FLRYQGVEF | 5 | SB | HLA-B1503 |
|  | E1-15 | 11 | FLRYQGVEF | 252 | WB | HLA-B1517 |
| 43 | E1-15 | 12 | LRYQGVEFI | 406 | WB | HLA-B1503 |
|  | E1-15 | 12 | LRYQGVEFI | 125 | WB | HLA-B2705 |
| 44 | E1-16 | 6 | PKKNCIVIY | 25 | SB | HLA-B1503 |
| 45 | E1-16 | 9 | NCIVIYGPA | 125 | WB | HLA-A6802 |
| 46 | E1-16 | 15 | GPANTGKSY | 326 | WB | HLA-B1502 |
|  | E1-16 | 15 | GPANTGKSY | 245 | WB | HLA-B3501 |
| 47 | E1-16 | 18 | NTGKSYFGM | 229 | WB | HLA-A2603 |
|  | E1-16 | 18 | NTGKSYFGM | 42 | SB | HLA-A6901 |
| 48 | E1-16 | 20 | GKSYFGMSL | 11 | SB | HLA-B1503 |
| 49 | E1-16 | 21 | KSYFGMSLI | 263 | WB | HLA-A0203 |
|  | E1-16 | 21 | KSYFGMSLI | 78 | WB | HLA-A3001 |
|  | E1-16 | 21 | KSYFGMSLI | 138 | WB | HLA-A3201 |
|  | E1-16 | 21 | KSYFGMSLI | 3 | SB | HLA-B1517 |
|  | E1-16 | 21 | KSYFGMSLI | 56 | WB | HLA-B5801 |
| 50 | E1-16 | 22 | SYFGMSLIH | 255 | WB | HLA-A2902 |
| 51 | E1-16 | 23 | YFGMSLIHF | 35 | SB | HLA-A2301 |
|  | E1-16 | 23 | YFGMSLIHF | 413 | WB | HLA-A2402 |
|  | E1-16 | 23 | YFGMSLIHF | 12 | SB | HLA-A2403 |
|  | E1-16 | 23 | YFGMSLIHF | 356 | WB | HLA-A2902 |
|  | E1-16 | 23 | YFGMSLIHF | 279 | WB | HLA-B1501 |
| 52 | E1-16 | 24 | FGMSLIHFL | 56 | WB | HLA-A0201 |
|  | E1-16 | 24 | FGMSLIHFL | 4 | SB | HLA-A0202 |
|  | E1-16 | 24 | FGMSLIHFL | 10 | SB | HLA-A0206 |
|  | E1-16 | 24 | FGMSLIHFL | 180 | WB | HLA-A0211 |
|  | E1-16 | 24 | FGMSLIHFL | 377 | WB | HLA-A0216 |
|  | E1-16 | 24 | FGMSLIHFL | 56 | WB | HLA-A6802 |
|  | E1-16 | 24 | FGMSLIHFL | 429 | WB | HLA-A6901 |
|  | E1-16 | 24 | FGMSLIHFL | 246 | WB | HLA-B3901 |
| 53 | E1-16 | 25 | GMSLIHFLQ | 187 | WB | HLA-A0202 |
|  | E1-16 | 25 | GMSLIHFLQ | 219 | WB | HLA-A2902 |
| 54 | E1-18 | 5 | ALDGNPISI | 55 | WB | HLA-A0201 |
|  | E1-18 | 5 | ALDGNPISI | 402 | WB | HLA-A0202 |
|  | E1-18 | 5 | ALDGNPISI | 379 | WB | HLA-A0203 |
|  | E1-18 | 5 | ALDGNPISI | 3 | SB | HLA-A0211 |
|  | E1-18 | 5 | ALDGNPISI | 8 | SB | HLA-A0212 |
|  | E1-18 | 5 | ALDGNPISI | 13 | SB | HLA-A0216 |
|  | E1-18 | 5 | ALDGNPISI | 21 | SB | HLA-A0219 |
|  | E1-18 | 5 | ALDGNPISI | 6 | SB | HLA-A0250 |
|  | E1-18 | 5 | ALDGNPISI | 68 | WB | HLA-A3201 |
| 55 | E1-18 | 9 | NPISIDRKH | 311 | WB | HLA-B3501 |
| 56 | E1-19 | 2 | VQLKCPPLL | 98 | WB | HLA-A0201 |
|  | E1-19 | 2 | VQLKCPPLL | 79 | WB | HLA-A0206 |
|  | E1-19 | 2 | VQLKCPPLL | 76 | WB | HLA-B1503 |
|  | E1-19 | 2 | VQLKCPPLL | 401 | WB | HLA-B3901 |
|  | E1-19 | 2 | VQLKCPPLL | 166 | WB | HLA-B4801 |
| 57 | E1-19 | 3 | QLKCPPLLI | 182 | WB | HLA-A0203 |
| 58 | E1-19 | 7 | PPLLITSNI | 430 | WB | HLA-B5301 |
| 59 | E1-20 | 1 | RWPYLHSRL | 133 | WB | HLA-A2402 |
|  | E1-20 | 1 | RWPYLHSRL | 24 | SB | HLA-A2403 |
| 60 | E1-20 | 2 | WPYLHSRLT | 267 | WB | HLA-B3501 |
|  | E1-20 | 2 | WPYLHSRLT | 88 | WB | HLA-B5401 |
| 61 | E1-20 | 4 | YLHSRLTVF | 275 | WB | HLA-A0202 |
|  | E1-20 | 4 | YLHSRLTVF | 91 | WB | HLA-A0203 |
|  | E1-20 | 4 | YLHSRLTVF | 479 | WB | HLA-A0211 |
|  | E1-20 | 4 | YLHSRLTVF | 210 | WB | HLA-A0250 |
|  | E1-20 | 4 | YLHSRLTVF | 27 | SB | HLA-B0801 |
|  | E1-20 | 4 | YLHSRLTVF | 28 | SB | HLA-B1501 |
|  | E1-20 | 4 | YLHSRLTVF | 59 | WB | HLA-B1502 |
|  | E1-20 | 4 | YLHSRLTVF | 8 | SB | HLA-B1503 |
|  | E1-20 | 4 | YLHSRLTVF | 303 | WB | HLA-B1517 |
| 62 | E1-21 | 4 | KNWKSFFSR | 7 | SB | HLA-A3101 |
| 63 | E1-21 | 6 | WKSFFSRTW | 93 | WB | HLA-B1503 |
| 64 | E1-21 | 8 | SFFSRTWSR | 230 | WB | HLA-A1101 |
|  | E1-21 | 8 | SFFSRTWSR | 7 | SB | HLA-A3101 |
|  | E1-21 | 8 | SFFSRTWSR | 13 | SB | HLA-A3301 |
|  | E1-21 | 8 | SFFSRTWSR | 56 | WB | HLA-A6801 |
| 65 | E1-21 | 9 | FFSRTWSRL | 443 | WB | HLA-A2301 |
|  | E1-21 | 9 | FFSRTWSRL | 192 | WB | HLA-A2403 |

^*^ Consensus conserved fragment number is taken from Table 1.

^**^ WB represents weak binder while SB represents strong binder
