# Supplementary material for: Sequence-based approach for rapid identification of cross-clade CD8+ T-cell vaccine candidates from all high-risk HPV strains
Source: 3 Biotech. 2016 Jan 27;6(1):39. doi: 10.1007/s13205-015-0352-z (PMC4729761; doi:10.1007/s13205-015-0352-z)
Supplement: Supplementary file 2 — Supplementary material 2 (DOCX 16 kb) [file 13205_2015_352_MOESM2_ESM.docx]

| Number of unique epitopes | Conserved fragment number* | Start position of epitope in the fragment | Epitope sequence | Affinity(nM) | Binding level** | HLA-allele  targeted |
| --- | --- | --- | --- | --- | --- | --- |
| 1 | E2-1 | 2 | ETLSQRLNV | 37 | SB | HLA-A6802 |
|  | E2-1 | 2 | ETLSQRLNV | 5 | SB | HLA-A6901 |
| 2 | E2-1 | 7 | RLNVCQDKI | 64 | WB | HLA-A0202 |
|  | E2-1 | 7 | RLNVCQDKI | 410 | WB | HLA-A0203 |
|  | E2-1 | 7 | RLNVCQDKI | 405 | WB | HLA-A0212 |
|  | E2-1 | 7 | RLNVCQDKI | 339 | WB | HLA-A0219 |
| 3 | E2-1 | 11 | CQDKILDHY | 149 | WB | HLA-A0101 |
|  | E2-1 | 11 | CQDKILDHY | 324 | WB | HLA-A8001 |
| 4 | E2-2 | 1 | ECAIFYKAR | 319 | WB | HLA-A6801 |
| 5 | E2-3 | 1 | QAIELQMAL | 446 | WB | HLA-A0206 |
|  | E2-3 | 1 | QAIELQMAL | 50 | WB | HLA-A6802 |
|  | E2-3 | 1 | QAIELQMAL | 132 | WB | HLA-A6901 |
|  | E2-3 | 1 | QAIELQMAL | 240 | WB | HLA-B3501 |
|  | E2-3 | 1 | QAIELQMAL | 189 | WB | HLA-B3901 |
| 6 | E2-3 | 3 | IELQMALES | 292 | WB | HLA-B4002 |
| 7 | E2-3 | 4 | ELQMALESL | 442 | WB | HLA-A0202 |
|  | E2-3 | 4 | ELQMALESL | 442 | WB | HLA-A0211 |
|  | E2-3 | 4 | ELQMALESL | 189 | WB | HLA-A0219 |
|  | E2-3 | 4 | ELQMALESL | 34 | SB | HLA-A0250 |
| 8 | E2-5 | 1 | CPESVSSTS | 424 | WB | HLA-B3501 |

**Supplementary Table 2:** Prediction of 9mer epitopes and their targeted alleles from the conserved consensus E2 protein fragment datasets of high-risk HPV strains

^*^ Consensus conserved fragment number is taken from Table 1.

^**^ WB represents weak binder while SB represents strong binder
