# Supplementary material for: Sequence-based approach for rapid identification of cross-clade CD8+ T-cell vaccine candidates from all high-risk HPV strains
Source: 3 Biotech. 2016 Jan 27;6(1):39. doi: 10.1007/s13205-015-0352-z (PMC4729761; doi:10.1007/s13205-015-0352-z)
Supplement: Supplementary file 3 — Supplementary material 3 (DOCX 15 kb) [file 13205_2015_352_MOESM3_ESM.docx]

| Number of unique epitopes | Conserved fragment number* | Start position of epitope in the fragment | Epitope sequence | Affinity(nM) | Binding level** | HLA-allele  targeted |
| --- | --- | --- | --- | --- | --- | --- |
| 1 | E6-2 | 1 | ALETSLHDI | 162 | WB | HLA-A0202 |
|  | E6-2 | 1 | ALETSLHDI | 300 | WB | HLA-A0203 |
|  | E6-2 | 1 | ALETSLHDI | 105 | WB | HLA-A0212 |
|  | E6-2 | 1 | ALETSLHDI | 10 | SB | HLA-A0250 |
| 2 | E6-3 | 1 | FYSKISEYR | 178 | WB | HLA-A2403 |
|  | E6-3 | 1 | FYSKISEYR | 102 | WB | HLA-A3101 |
|  | E6-3 | 1 | FYSKISEYR | 46 | SB | HLA-A3301 |
|  | E6-3 | 1 | FYSKISEYR | 12 | SB | HLA-A6801 |
| 3 | E6-3 | 2 | YSKISEYRH | 120 | WB | HLA-B1517 |
| 4 | E6-3 | 3 | SKISEYRHY | 430 | WB | HLA-A2602 |
|  | E6-3 | 3 | SKISEYRHY | 6 | SB | HLA-B1503 |
| 5 | E6-5 | 1 | KKRFHNIAG | 157 | WB | HLA-A3001 |

**Supplementary Table 3:** Prediction of 9mer epitopes and their targeted alleles from the conserved consensus E6 protein fragment datasets of high-risk HPV strains

^*^ Consensus conserved fragment number is taken from Table 1.

^**^ WB represents weak binder while SB represents strong binder
