# Supplementary material for: Sequence-based approach for rapid identification of cross-clade CD8+ T-cell vaccine candidates from all high-risk HPV strains
Source: 3 Biotech. 2016 Jan 27;6(1):39. doi: 10.1007/s13205-015-0352-z (PMC4729761; doi:10.1007/s13205-015-0352-z)
Supplement: Supplementary file 4 — Supplementary material 4 (DOCX 14 kb) [file 13205_2015_352_MOESM4_ESM.docx]

| Number of unique epitopes | Conserved fragment number* | Start position of epitope in the fragment | Epitope sequence | Affinity(nM) | Binding level** | HLA-asllele  targeted |
| --- | --- | --- | --- | --- | --- | --- |
| 1 | E7-1 | 2 | TLQDIVLDL | 47 | SB | HLA-A0201 |
|  | E7-1 | 2 | TLQDIVLDL | 46 | SB | HLA-A0202 |
|  | E7-1 | 2 | TLQDIVLDL | 54 | WB | HLA-A0203 |
|  | E7-1 | 2 | TLQDIVLDL | 89 | WB | HLA-A0211 |
|  | E7-1 | 2 | TLQDIVLDL | 70 | WB | HLA-A0212 |
|  | E7-1 | 2 | TLQDIVLDL | 116 | WB | HLA-A0216 |
|  | E7-1 | 2 | TLQDIVLDL | 73 | WB | HLA-A0219 |
|  | E7-1 | 2 | TLQDIVLDL | 4 | SB | HLA-A0250 |
| 2 | E7-2 | 1 | LQQLLMGTL | 468 | WB | HLA-A0206 |
|  | E7-2 | 1 | LQQLLMGTL | 482 | WB | HLA-B1501 |

**Supplementary Table 4:** Prediction of 9mer epitopes and their targeted alleles from the conserved consensus E7 protein fragment datasets of high-risk HPV strains

^*^ Consensus conserved fragment number is taken from Table 1.

^**^ WB represents weak binder while SB represents strong binder
