# Supplementary material for: Sequence-based approach for rapid identification of cross-clade CD8+ T-cell vaccine candidates from all high-risk HPV strains
Source: 3 Biotech. 2016 Jan 27;6(1):39. doi: 10.1007/s13205-015-0352-z (PMC4729761; doi:10.1007/s13205-015-0352-z)
Supplement: Supplementary file 5 — Supplementary material 5 (DOCX 35 kb) [file 13205_2015_352_MOESM5_ESM.docx]

**Multiple sequence alignment of E1 Protein dataset from all high-risk HPV strain:**

HPV51:gi|137655|sp|P26544.1| MDC-EGTE-DEGAGCNGWFFVEAIVEKKTGDNVSDDEDENADDTG-SDLI 47 HPV89:gi|6970430|dbj|BAA90737.1| MDS-EGTE-DEGAGCTGWFYVEAVVDKKTGDNISDDEEEDTNDTG-SDII 47

HPV69:gi|6970421|dbj|BAA90729.1| MDC-EGTD-GEGLGCTGWFSVEAIVEKHTGETISEDEIEYSSDTG-SDLI 47

HPV56:tr|A9XCP9|A9XCS0 MASPEGTD-GEGKGCCGWFEVEAIVEKKTGDKISDDESDEEDEID-TDLD 48

HPV73:gi|1491695|emb|CAA63884.1| MAD--SGN-WEGR-CTGWFNVEAIVERKTGDPIPEDENYDGGDTDESEMG 46

HPV31:gi|137648|sp|P17382.1| MADPAGTD-GEGTGCNGWFYVEAVIDRQTGDNISEDENEDSSDTG-EDMV 48

HPV35:gi|337238518|gb|AEI61432.1| MADPAGTDEGEGTGCNGWFFVEAVVSRRTGDPVSEDENEDNCDRG-EDMV 49

HPV16:gi|242347746|gb|ACS92694.1| MADPAGTNGEEGTGCNGWFYVEAVVEKKTGDAISDDENENDSDTG-EDLV 49

HPV33:gi|218931425|gb|ACL12328.1| MADPEGTN-GAGMGCTGWLEVEAVIERRTGDNISEDEDETADDSG-TDLL 48

HPV58:gi|260169933|gb|ACX32371.1| MDDPEGTN-GVGAGCTGWFEVEAVIERRTGDNISDDEDETADDSG-TDLI 48

HPV52:gi|260169919|gb|ACX32359.1| MEDPEGTE-GEREGCTGWFEVEAIIEKQTGDNISEDEDENAYDSG-TDLI 48

HPV18:gi|60978|emb|CAA28666.1| MADPEGTD-GEGTGCNGWFYVQAIVDKKTGDVISDDEDENATDTG-SDMV 48

HPV45:gi|145968373|gb|ABP99898.1| MADPEGTD-GEGTGCNGWFFVETIVEKKTGDVISDDEDETATDTG-SDMV 48

HPV59:gi|218931434|gb|ACL12336.1| MADSEGTD-GEGTGCNGWFFVQAIVDKKTGDKISDDEDENATDTG-SDLV 48

HVP39:gi|137651|sp|P24829.1| MANREGTD-GDGSGCNGWFLVQAIVDKQTGDTVSEDEDENATDTG-SDLA 48

HPV68:gi|260169942|gb|ACX32379.1| MANCEGTD-GDGTGCNGWFFVQAIVDKQAGDTVSEDEDENATDTG-SDMV 48

* . : * **: *::::.:::*: :.:** : . ::

HPV51:gi|137655|sp|P26544.1| NFIDSETSICSQAEQETARALFQAQELQANKEAVHQLKRKFLVSPRSSPL 97

HPV89:gi|6970430|dbj|BAA90737.1| DFIDTSNSICSQAERETAQALLQVQETQAHKEAVQHLKRKFLGSPRSSPL 97

HPV69:gi|6970421|dbj|BAA90729.1| GFIDDSN-ISDGAEQQVAQALFQAQETQANKKAVRALKRKLLGS-QNSPL 95

HPV56:tr|A9XCP9|A9XCS0 GFIDDSYIQNIQADAETGQQLLQVQTAHADKQTLQKLKRKYIAS----PL 94

HPV73:gi|1491695|emb|CAA63884.1| DFIDNAHIPNIYAQQEIAQALYQSQQANADNEAIRVLKRKFTGSPGGSPD 96

HPV31:gi|137648|sp|P17382.1| DFIDNCNVYNNQAEAETAQALFHAQEAEEHAEAVQVLKRKYVGSPLSDI- 97

HPV35:gi|337238518|gb|AEI61432.1| DFINDTDILNIQAETETAQALFHAQEEQTHKEAVQVLKRKYASSPLSSV- 98

HPV16:gi|242347746|gb|ACS92694.1| DFIVNDNDYLTQAETETAHALFTAQEAKQHRDAVQVLKRKYLGSPLSDI- 98

HPV33:gi|218931425|gb|ACL12328.1| EFIDDSMENSIQADTEAARALFNIQEGEDDLNAVCALKRKFAACSQSAA- 97

HPV58:gi|260169933|gb|ACX32371.1| EFIDDSVQSTTQAEAEAARALFNVQEGVDDINAVCALKRKFAACSESAV- 97

HPV52:gi|260169919|gb|ACX32359.1| DFIDDSNINNEQAEHEAARALFNAQEGEDDVHAVSAVKRKFTSSPESAG- 97

HPV18:gi|60978|emb|CAA28666.1| DFIDTQGTFCEQAELETAQALFHAQEVHNDAQVLHVLKRKFAGGSTENSP 98

HPV45:gi|145968373|gb|ABP99898.1| DFIDTQLSICEQAEQETAQALFHAQEVQNDAQVLHLLKRKFAGGSKENSP 98

HPV59:gi|218931434|gb|ACL12336.1| DFIDDTTTICVQAERETAQALFNVQEAQRDAREMHVLKRKFG-CSIENS- 96

HVP39:gi|137651|sp|P24829.1| DFIDDSTDICVQAERETAQVLLHMQEAQRDAQAVRALKRKYTDSSGDTRP 98

HPV68:gi|260169942|gb|ACX32379.1| DFIDDATDICIQAERETAQVLLNMQQAQRDAQTVRALKRKYTDS-IESSP 97

** *: : .: * * . : :***

HPV51:gi|137655|sp|P26544.1| GDITNQNNTHSHS-------QANESQVKRRLLDSYPDSGYGNTQVETVEA 140

HPV89:gi|6970430|dbj|BAA90737.1| KDITNQN-TQSNSQQ--QPKQANLQHGKRRLLDSYPDSGYGNTQVETVEG 144

HPV69:gi|6970421|dbj|BAA90729.1| QDITNQSNSQQST------DEVNNLQAKRRAVDSVPDSGYGYTEVETLT- 138

HPV56:tr|A9XCP9|A9XCS0 RDISNQQTVCREG------------VKRRLILSDLQDSGYGNT-LETLET 131

HPV73:gi|1491695|emb|CAA63884.1| MKRDEFIDKQLSPQINVLSISSGRSTSKRRLFEEQ-DSGYGNTEVETYET 145

HPV31:gi|137648|sp|P17382.1| ---SSCVDYNISPRLKAICIENNSKTAKRRLFELP-DSGYGNTEVETQQM 143

HPV35:gi|337238518|gb|AEI61432.1| ---SLCVNNNISPRLKAICIENKNTAAKRRLFELP-DSGYGNSEVEIQQI 144

HPV16:gi|242347746|gb|ACS92694.1| ---SGCVDNNISPRLKAICIEKQSRAAKRRLFESE-DSGYGNTEVETQQM 144

HPV33:gi|218931425|gb|ACL12328.1| ---EDVVDRAANPCRTSINKNKECTYRKRKIDELE-DSGYGNTEVETQQM 143

HPV58:gi|260169933|gb|ACX32371.1| ---EDCVDRAANVCVSWKYKNKECTHRKRKIIELE-DSGYGNTEVETEQM 143

HPV52:gi|260169919|gb|ACX32359.1| ---QDGVEKHGSPRAKHICVNTECVLPKRKPCQVE-DSGYGNSEVEAQQM 143

HPV18:gi|60978|emb|CAA28666.1| LGERLEVDTELSPRLQEISLNSGQKKAKRRLFTIS-DSGYGCSEVEATQI 147

HPV45:gi|145968373|gb|ABP99898.1| LGEQLSVETDLSPRLQEISLNSGHKKAKRRLFTIS-DSGYGCSEVEAAET 147

HPV59:gi|218931434|gb|ACL12336.1| -SEKAAAGKKAKSPLQEISVNVNHPKVKRRLLTVP-DSGYGYSEVEMLET 144

HVP39:gi|137651|sp|P24829.1| YGKKVGRNTRGT--LQEISLNVSSTQATQTVYSVP-DSGYGNMEVETAEV 145

HPV68:gi|260169942|gb|ACX32379.1| LAKSP---------LQELSINVSSTQARQPAYSVP-DSGYGNMEVETHS- 136

: ***** :*

HPV51:gi|137655|sp|P26544.1| TLQVDGQHGGSQ----NSVCSSGGGS----VMDVETT-ESCANVELN--- 178

HPV89:gi|6970430|dbj|BAA90737.1| PLQVDGQNDGSQ----HSMCSGGGSSDRSTEIDLETN-ENATNVGLN--- 186

HPV69:gi|6970421|dbj|BAA90729.1| PVQVDKHNEQNG----DSVCSQGGSS--GSVSDMEVD-IGAQASSVT--- 178

HPV56:tr|A9XCP9|A9XCS0 PEQVDEEVQGRG----CGNTQNGGSQNSTYSNNSEDS-VIHMDIDRNNET 176

HPV73:gi|1491695|emb|CAA63884.1| EVPGLGAGVGCL----QNVNEEGNQIVSPRESSSGSSSISNMDIETES-T 190

HPV31:gi|137648|sp|P17382.1| VQVEE-QQTTLS-----CNGSDG----------------THSERE--NET 169

HPV35:gi|337238518|gb|AEI61432.1| QQVEG-HDTVEQ-----CSMGSGDSIT-----------SSSDERH--EET 175

HPV16:gi|242347746|gb|ACS92694.1| LQVEGRHETETP-----CSQYSGGSGGGCSQYSSGSGGEGVSERHTICQT 189

HPV33:gi|218931425|gb|ACL12328.1| VQQVESQNGDTN-----LNDLESSGVGDD----SEVSCETNVDSCEN--V 182

HPV58:gi|260169933|gb|ACX32371.1| AHQVESQNGDAD-----LNDSQSSGVGAS----SDVSSETDVDSCNT--V 182

HPV52:gi|260169919|gb|ACX32359.1| ADQVDGQNGDWQ-----SNSSQSSGVGASN---SDVSCTSIEDNEENSNR 185

HPV18:gi|60978|emb|CAA28666.1| -QVTTNGEHGGNVCSGGSTEAIDNGGTEGNNSSVDGTSDNSNIENVNPQC 196

HPV45:gi|145968373|gb|ABP99898.1| -QVTVN------------TNAENGGSVHSTQSSGGDSSDN--AENVDPQC 182

HPV59:gi|218931434|gb|ACL12336.1| -QVTVEN----------TGNGDSNGSVCSDSQIDCSDSSNMDVENIVPTS 183

HVP39:gi|137651|sp|P24829.1| EEVTVATNTN------GDAEGEHGGSVREECSSVDSAIDS---ENQDPKS 186

HPV68:gi|260169942|gb|ACX32379.1| -EVTVATNTNG---ADGEDEGENGDSIREDCSSVDSAIDS---ENQDPKS 179

HPV51:gi|137655|sp|P26544.1| ---SICEVLKSSNAKATLMAKFKELYGISYNELVRVFKSDKTCCIDWVCA 225

HPV89:gi|6970430|dbj|BAA90737.1| ---SICAVLKCSNAKAMFMAKFKELYGVSYNELVRVFKSDKTCCTDWVCA 233

HPV69:gi|6970421|dbj|BAA90729.1| ---KICELLKCSNVKAALLSKFKTVYGVSYTELVRVFKSDKTCCSDWVCA 225

HPV56:tr|A9XCP9|A9XCS0 PTQQLQDLFKSSNLQGKLYYKFKEVYGIPFSELVRTFKSDSTCCNDWICA 226

HPV73:gi|1491695|emb|CAA63884.1| PITDITNLLQRNNAKAALLAKFKEVYGLSYMELVRPYKSDKTHCQDWVCA 240

HPV31:gi|137648|sp|P17382.1| PTRNILQVLKTSNGKAAMLGKFKELYGVSFMELIRPFQSNKSTCTDWCVA 219

HPV35:gi|337238518|gb|AEI61432.1| PTRDIIQILKCSNANAAMLAKFKELFGISFTELIRPFKSDKSTCTDWCVA 225

HPV16:gi|242347746|gb|ACS92694.1| PLTNILNVLKTSNAKAAMLAKFKELYGVSFTELVRPFKSNKSTCCDWCIA 239

HPV33:gi|218931425|gb|ACL12328.1| TLQEISNVLHSSNTKANILYKFKEAYGISFMELVRPFKSDKTSCTDWCIT 232

HPV58:gi|260169933|gb|ACX32371.1| PLQNISNILHNSNTKATLLYKFKEAYGVSFMELVRPFKSDKTSCTDWCIT 232

HPV52:gi|260169919|gb|ACX32359.1| TLKSIQNIMCENSIKTTVLFKFKETYGVSFMELVRPFKSNRSSCTDWCII 235

HPV18:gi|60978|emb|CAA28666.1| TIAQLKDLLKVNNKQGAMLAVFKDTYGLSFTDLVRNFKSDKTTCTDWVTA 246

HPV45:gi|145968373|gb|ABP99898.1| SITELKELLQASNKKAAMLAVFKDIYGLSFTDLVRNFKSDKTTCTDWVMA 232

HPV59:gi|218931434|gb|ACL12336.1| PTNQLLQLLHSKNKKAAMYAKFKELYGLSFQDLVRTFKSDRTTCSDWVTA 233

HVP39:gi|137651|sp|P24829.1| PTAQIKLLLQSNNKKAAMLTQFKETYGLSFTDLVRTFKSDKTTCTDWVAA 236

HPV68:gi|260169942|gb|ACX32379.1| PTTQLKVLLQCNNKKAAMLTEFKKVYGLSFNDLVRTFKSDKTTCTDWVAA 229

.: :: .. : . ** :*:.: :*:* ::*: : * **

HPV51:gi|137655|sp|P26544.1| LFGVSPMVAENLKTLIKPFCMYYHIQCLSCDWGTIVLMLIRFSCAKNRTT 275

HPV89:gi|6970430|dbj|BAA90737.1| LFGVSPMVAENLKTLIQPFCMYYHIQCLSCDWGTIVLLLARFTCAKNRLT 283

HPV69:gi|6970421|dbj|BAA90729.1| AFGVAGSVAESLKTLIQPYCLYYHIQCLTCNWGVLPLMLIRFTCAKNRAT 275

HPV56:tr|A9XCP9|A9XCS0 IFGVNETLAEALKTIIKPHCMYYHMQCLTCTWGVIVMMLIRYTCGKNRKT 276

HPV73:gi|1491695|emb|CAA63884.1| VFGVIPSLAESLKSLLTQYCMYIHLQCLTCTWGIIVLVLVRFKCNKNRLT 290

HPV31:gi|137648|sp|P17382.1| AFGVTGTVAEGFKTLLQPYCLYCHLQSLACSWGMVMLMLVRFKCAKNRIT 269

HPV35:gi|337238518|gb|AEI61432.1| AFGIAPSVAESLKTLIKPYCLYVHIQCLSCSWGMVILALLRFKCAKNRTT 275

HPV16:gi|242347746|gb|ACS92694.1| AFGLTPSIADSIKTLLQQYCLYLHIQSLACSWGMVVLLLVRYKCGKNRET 289

HPV33:gi|218931425|gb|ACL12328.1| GYGISPSVAESLKVLIKQHSLYTHLQCLTCDRGIIILLLIRFRCSKNRLT 282

HPV58:gi|260169933|gb|ACX32371.1| GYGISPSVAESLKVLIKQHSIYTHLQCLTCDRGIILLLLIRFKCSKNRLT 282

HPV52:gi|260169919|gb|ACX32359.1| GMGVTPSVAEGLKVLIQPYSIYAHSQCLTCDRGVLILLLIRFKCGKNRLT 285

HPV18:gi|60978|emb|CAA28666.1| IFGVNPTIAEGFKTLIQPFILYAHIQCLDCKWGVLILALLRYKCGKSRLT 296

HPV45:gi|145968373|gb|ABP99898.1| IFGVNPTVAEGFKTLIKPATLYAHIQCLDCKWGVLILALLRYKCGKNRLT 282

HPV59:gi|218931434|gb|ACL12336.1| IFGVNPTVAEGFKTLIQPYVLYAHIQCLDCAWGVVILALLRYKCGKNRIT 283

HVP39:gi|137651|sp|P24829.1| IFGVHPTIAEGFKTLINKYALYTHIQSLDTKQGVLILMLIRYTCGKNRVT 286

HPV68:gi|260169942|gb|ACX32379.1| IFGVNPTIAEGFKTLIKQYALYTHIQCLDTKNGILILMLIRYKCGKNRIT 279

*: :*: :* :: :* * *.* * : : * *: * *.* *

HPV51:gi|137655|sp|P26544.1| IAKCLSTLVNIPQSQMFIEPPKLRSTPVALYFYRTGISNISNTYGETPEW 325

HPV89:gi|6970430|dbj|BAA90737.1| IAKCLGTLVNIPQSQMFIEPPKLRSTAVALYFYRTGISNISSTYGETPEW 333

HPV69:gi|6970421|dbj|BAA90729.1| IKKCLCTLLNVPDTQLLIEPPKLRSTAVALYFYKTGLSNISETHGDTPEW 325

HPV56:tr|A9XCP9|A9XCS0 IAKALSSILNVPQEQMLIQPPKIRSPAVALYFYKTAMSNISDVYGDTPEW 326

HPV73:gi|1491695|emb|CAA63884.1| VQKLLSSLLNVTQERMLIEPPRLRSTPCALYWYRTSLSNISEIVGDTPEW 340

HPV31:gi|137648|sp|P17382.1| IEKLLEKLLCISTNCMLIQPPKLRSTAAALYWYRTGMSNISDVYGETPEW 319

HPV35:gi|337238518|gb|AEI61432.1| IEKLLSKLLCISAASMLIQPPKLRSTPAALYWFKTAMSNISEVDGETPEW 325

HPV16:gi|242347746|gb|ACS92694.1| IEKLLSKLLCVSPMCMMIEPPKLRSTAAALYWYKTGISNISEVYGDTPEW 339

HPV33:gi|218931425|gb|ACL12328.1| VAKLMSNLLSIPETCMVIEPPKLRSQTCALYWFRTAMSNISDVQGTTPEW 332

HPV58:gi|260169933|gb|ACX32371.1| VAKLMSNLLSIPETCMIIEPPKLRSHACALYWFRTAMSNISDVQGTTPEW 332

HPV52:gi|260169919|gb|ACX32359.1| VSKLMSQLLNIPETHMVIEPPKLRSATCALYWYRTGLSNISQVYGTTPEW 335

HPV18:gi|60978|emb|CAA28666.1| VAKGLSTLLHVPETCMLIQPPKLRSSVAALYWYRTGISNISEVMGDTPEW 346

HPV45:gi|145968373|gb|ABP99898.1| VAKGLSTLLQVPETCMLIEPPKLRSSVAALYWYRTGISNISEVSGDTPEW 332

HPV59:gi|218931434|gb|ACL12336.1| VAKGLSTLLHVPDTCMLIEPPKLRSGVAALYWYRTGMSNISEVIGETPEW 333

HVP39:gi|137651|sp|P24829.1| VGKGLSTLLHVPESCMLLEPPKLRSPVAALYWYRTGISNISVVTGDTPEW 336

HPV68:gi|260169942|gb|ACX32379.1| VGKGLSTLLHVPDSCMLLQPPKLRSPVAALYWYRTGISNISEVCGDTPEW 329

: * : :: :. :.::**::** ***:::*.:**** * ****

HPV51:gi|137655|sp|P26544.1| ITRQTQLQHSFEDSTFELSQMVQWAFDHEVLDDSEIAFHYAQLADIDSNA 375

HPV89:gi|6970430|dbj|BAA90737.1| ITRQTQLQHSFDDSTFELSQMVQWAFDHDVVDDSEIAFYYAQLADTDSNA 383

HPV69:gi|6970421|dbj|BAA90729.1| IVRQTQLEHSFEDTIFDLSKMVQWAFDHDITDDSEIAFKYAQLADIESNA 375

HPV56:tr|A9XCP9|A9XCS0 IQRQTQLQHSLQDSQFELSKMVQWAFDNEVTDDSQIAFQYAQLADVDSNA 376

HPV73:gi|1491695|emb|CAA63884.1| IKRQTLVQHSLDDSQFDLSQMIQWAFDNDITDDCEIAYKYALLGNVDSNA 390

HPV31:gi|137648|sp|P17382.1| IERQTVLQHSFNDTTFDLSQMVQWAYDNDVMDDSEIAYKYAQLADSDSNA 369

HPV35:gi|337238518|gb|AEI61432.1| IQRQTVLQHSFNDAIFDLSEMVQWAYDNDFIDDSDIAYKYAQLAETNSNA 375

HPV16:gi|242347746|gb|ACS92694.1| IQRQTVLQHSFNDCTFELSQMVQWAYDNDIVDDSEIAYKYAQLADTNSNA 389

HPV33:gi|218931425|gb|ACL12328.1| IDRLTVLQHSFNDNIFDLSEMVQWAYDNELTDDSDIAYYYAQLADSNSNA 382

HPV58:gi|260169933|gb|ACX32371.1| IDRLTVLQHSFNDDIFDLSEMIQWAYDNDITDDSDIAYKYAQLADVNSNA 382

HPV52:gi|260169919|gb|ACX32359.1| IEHQTVLQHSFDNSIFDFGEMVQWAYDHDITDDSDIAYKYAQLADVNSNA 385

HPV18:gi|60978|emb|CAA28666.1| IQRLTIIQHGIDDSNFDLSEMVQWAFDNELTDESDMAFEYALLADSNSNA 396

HPV45:gi|145968373|gb|ABP99898.1| IQRLTIIQHGIDDSNFDLSDMVQWAFDNDLTDESDMAFQYAQLADCNSNA 382

HPV59:gi|218931434|gb|ACL12336.1| IQRLTIIQHGVDDSVFDLSEMIQWAFDNDLTDESDIAYEYALIADSNSNA 383

HVP39:gi|137651|sp|P24829.1| IQRLTVIQHGIDDSVFDLSDMVQWAFDNEYTDESDIAFNYAMLADCNSNA 386

HPV68:gi|260169942|gb|ACX32379.1| IKRLTIIQHGIDDSVFDLSDMVQWAFDNELTDESDIAFSYAMLADCNSNA 379

* : * ::*..:: *::..*:***:*:: *:.::*: ** :.: :***

HPV51:gi|137655|sp|P26544.1| AAFLKSNCQAKYVKDCGTMARHYKRAQRKSLSMSAWIRYRCDRAKDGGNW 425

HPV89:gi|6970430|dbj|BAA90737.1| AAFLKSNCQAKYVKDCGTMTRHYKRAQRKSLTMSAWIRYRCDKVQDGGNW 433

HPV69:gi|6970421|dbj|BAA90729.1| AAFLKSNCQAKYVKDCATMTRHYKRAQKRSMGMSQWLQHRCSKIEDGGTW 425

HPV56:tr|A9XCP9|A9XCS0 QAFLKSNMQAKYVKDCGIMCRHYKRAQQQQMNMCQWIKHICSKTDEGGDW 426

HPV73:gi|1491695|emb|CAA63884.1| AAFLKSNAQAKYVKDCGTMCRHYKAAERKQMSMAQWIQHRCDLTNDGGNW 440

HPV31:gi|137648|sp|P17382.1| CAFLKSNSQAKIVKDCGTMCRHYKRAEKRQMSMGQWIKSRCDKVSDEGDW 419

HPV35:gi|337238518|gb|AEI61432.1| CAFLKSNSQAKIVKDCATMCRHYKRAEKREMTMSQWIKRRCEKVDDDGDW 425

HPV16:gi|242347746|gb|ACS92694.1| SAFLKSNSQAKIVKDCATMCRHYKRAEKKQMSMSQWIKYRCDRVDDGGDW 439

HPV33:gi|218931425|gb|ACL12328.1| AAFLKSNSQAKIVKDCGIMCRHYKKAEKRKMSIGQWIQSRCEKTNDGGNW 432

HPV58:gi|260169933|gb|ACX32371.1| AAFLRSNAQAKIVKDCGVMCRHYKRAEKRGMTMGQWIQSRCEKTNDGGNW 432

HPV52:gi|260169919|gb|ACX32359.1| AAFLKSNSQAKIVKDCATMCRHYKRAERKHMNIGQWIQYRCDRIDDGGDW 435

HPV18:gi|60978|emb|CAA28666.1| AAFLKSNCQAKYLKDCATMCKHYRRAQKRQMNMSQWIRFRCSKIDEGGDW 446

HPV45:gi|145968373|gb|ABP99898.1| AAFLKSNCQAKYLKDCAVMCRHYKRAQKRQMNMSQWIKYRCSKIDEGGDW 432

HPV59:gi|218931434|gb|ACL12336.1| AAFLKSNCQAKYLKDCAVMCRHYKRAQKRQMSMSQWIKWRCDKIEEGGDW 433

HVP39:gi|137651|sp|P24829.1| AAFLKSNCQAKYVKDCATMCKHYKRAQKRQMSMSQWIKFRCSKCDEGGDW 436

HPV68:gi|260169942|gb|ACX32379.1| AAFLKSNCQAKYVKDCATMCRHYKRAQKRQMSMPQWIKFRCSKCDEGGDW 429

***:** *** :***. * :**: *::: : : *:: *. .: * *

HPV51:gi|137655|sp|P26544.1| REIAKFLRYQGVNFMSFIQMFKQFLKGTPKHNCIVIYGPPNTGKSLFAMS 475

HPV89:gi|6970430|dbj|BAA90737.1| REIAKFLRYQGINFMYFIQTFKLFLKGTPKHNCIVIQGPPNTGKSQFAMS 483

HPV69:gi|6970421|dbj|BAA90729.1| KDIARFLRYQNVNFIYFLQVLKQFLKGTPKHNCIVIYGPPNTGKSQFAMS 475

HPV56:tr|A9XCP9|A9XCS0 KPIVQFLRYQGVDFISFLSYFKLFLQGTPKHNCLVLCGPPNTGKSCFAMS 476

HPV73:gi|1491695|emb|CAA63884.1| KDIVLFLRYQNVEFMPFLITLKQFLKGIPKQNCIVLYGPPDTGKSHFGMS 490

HPV31:gi|137648|sp|P17382.1| RDIVKFLRYQQIEFVSFLSALKLFLKGVPKKNCILIHGAPNTGKSYFGMS 469

HPV35:gi|337238518|gb|AEI61432.1| RDIVRFLRYQQVDFVAFLSALKNFLHGVPKKNCILIYGAPNTGKSLFGMS 475

HPV16:gi|242347746|gb|ACS92694.1| KQIVMFLRYQGVEFMSFLTALKRFLQGIPKKNCILLYGAANTGKSLFGMS 489

HPV33:gi|218931425|gb|ACL12328.1| RPIVQLLRYQNIEFTAFLGAFKKFLKGIPKKSCILICGPANTGKSYFGMS 482

HPV58:gi|260169933|gb|ACX32371.1| RPIVQFLRYQNIEFTAFLVAFKQFLQGVPKKSCMLLCGPANTGKSYFGMS 482

HPV52:gi|260169919|gb|ACX32359.1| RPIVRFLRYQDIEFTAFLDAFKKFLKGIPKKNCLVLYGPANTGKSYFGMS 485

HPV18:gi|60978|emb|CAA28666.1| RPIVQFLRYQQIEFITFLGALKSFLKGTPKKNCLVFCGPANTGKSYFGMS 496

HPV45:gi|145968373|gb|ABP99898.1| RPIVQFLRYQGVEFISFLRALKEFLKGTPKKNCILLYGPANTGKSYFGMS 482

HPV59:gi|218931434|gb|ACL12336.1| KPIVQFLRYQGVEFITFLCALKDFLKGTPKRNCIVLCGPANTGKSYFGMS 483

HVP39:gi|137651|sp|P24829.1| RPIVQFLRYQGIEFISFLCALKEFLKGTPKKNCIVIYGPANTGKSHFCMS 486

HPV68:gi|260169942|gb|ACX32379.1| RPIVQFLRYQGLEFITFLCALKDFLKGTPKRNCIGIHGPPNTGKSYFCMS 479

: *. :**** ::* *: :* **:* **:.*: : *..:**** * **

HPV51:gi|137655|sp|P26544.1| LMKFMQGSIISYVNSGSHFWLQPLEDAKIALLDDATYGCWTYIDQYLRNF 525

HPV89:gi|6970430|dbj|BAA90737.1| LIRFLQGCVISYVNSGSHFWLQPLEDAKVALLDDATYGCWTYIDQYLRNF 533

HPV69:gi|6970421|dbj|BAA90729.1| FIKFVQGSVISYVNSNSHFWLQPLEDAKVALLDDATYGCWLYIDKYLRNF 525

HPV56:tr|A9XCP9|A9XCS0 LIKFFQGSVISFVNSQSHFWLQPLDNAKLGLLDDATEICWKYIDDYLRNL 526

HPV73:gi|1491695|emb|CAA63884.1| LIKFIQGVVISYVNSTSHFWLSPLADAKMALLDDATPGCWTYIDKYLRNA 540

HPV31:gi|137648|sp|P17382.1| LISFLQGCIISYANSKSHFWLQPLADAKIGMLDDATTPCWHYIDNYLRNA 519

HPV35:gi|337238518|gb|AEI61432.1| LMHFLQGAIISYVNSKSHFWLQPLYDAKIAMLDDATSPCWAYIDQYLRNA 525

HPV16:gi|242347746|gb|ACS92694.1| LMKFLQGSVICFVNSKSHFWLQPLADAKIGMLDDATVPCWNYIDDNLRNA 539

HPV33:gi|218931425|gb|ACL12328.1| LIQFLKGCVISCVNSKSHFWLQPLSDAKIGMIDDVTPISWTYIDDYMRNA 532

HPV58:gi|260169933|gb|ACX32371.1| LIHFLKGCIISYVNSKSHFWLQPLPDAKLGMIDDVTAISWTYIDDYMRNA 532

HPV52:gi|260169919|gb|ACX32359.1| LIRFLSGCVISYVNSKSHFWLQPLTDAKVGMIDDVTPICWTYIDDYMRNA 535

HPV18:gi|60978|emb|CAA28666.1| FIHFIQGAVISFVNSTSHFWLEPLTDTKVAMLDDATTTCWTYFDTYMRNA 546

HPV45:gi|145968373|gb|ABP99898.1| FIHFLQGAIISFVNSNSHFWLEPLADTKVAMLDDATHTCWTYFDNYMRNA 532

HPV59:gi|218931434|gb|ACL12336.1| LLHFLQGTVISHVNSNSHFWLEPLTDAKLAMLDDATDSCWTYFDTYMRNA 533

HVP39:gi|137651|sp|P24829.1| LMHFLQGTVISYVNSTSHFWLEPLADAKLAMLDDATGTCWSYFDNYMRNA 536

HPV68:gi|260169942|gb|ACX32379.1| LIHFLQGTIISYVNSASHFWLEPLADAKIAMLDDATGTCWSYFDNYMRNA 529

:: *..* :*. .** *****.** ::*:.::**.* .* *:* :**

HPV51:gi|137655|sp|P26544.1| LDGNPCSIDRKHRSLIQLVCPPLLITSNINPQEDANLMYLHTRVTVLKFL 575

HPV89:gi|6970430|dbj|BAA90737.1| LNGNPCSIDRKHRSLLQIVCPPLLITSNINPKEDPNLMYLHSRVTVFQFL 583

HPV69:gi|6970421|dbj|BAA90729.1| LDGNPCCIDRKHRSLIQVRCPPLIITSNINPQDDNSLMYLHSRVTVIPFP 575

HPV56:tr|A9XCP9|A9XCS0 VDGNPISLDRKHKQLVQIKCPPLLITTNINPMLDAKLRYLHSRMLVFQFQ 576

HPV73:gi|1491695|emb|CAA63884.1| LDGNPICLDRKHKNLLQVKCPPLLITSNTNPKADDTWKYLHSRIKVFTFL 590

HPV31:gi|137648|sp|P17382.1| LDGNPVSIDVKHKALMQLKCPPLLITSNINAGKDDRWPYLHSRLVVFTFP 569

HPV35:gi|337238518|gb|AEI61432.1| LDGNPISLDVKHKALVQLKCPPLLITSNINAGKDDRWPYLHSRVVVFTFH 575

HPV16:gi|242347746|gb|ACS92694.1| LDGNLVSMDVKHRPLVQLKCPPLLITSNINAGTDSRWPYLHNRLVVFTFP 589

HPV33:gi|218931425|gb|ACL12328.1| LDGNEISIDVKHRALVQLKCPPLLLTSNTNAGTDSRWPYLHSRLTVFEFK 582

HPV58:gi|260169933|gb|ACX32371.1| LDGNDISIDVKHRALVQLKCPPLIITSNTNAGKDSRWPYLHSRLTVFEFN 582

HPV52:gi|260169919|gb|ACX32359.1| LDGNDISVDVKHRALVQIKCPPLILTTNTNAGTDPGWPYLHSRLVVFHFK 585

HPV18:gi|60978|emb|CAA28666.1| LDGNPISIDRKHKPLIQLKCPPILLTTNIHPAKDNRWPYLESRITVFEFP 596

HPV45:gi|145968373|gb|ABP99898.1| LDGNPISIDRKHKPLLQLKCPPILLTSNIHPAKDNKWPYLESRVTVFTFP 582

HPV59:gi|218931434|gb|ACL12336.1| LDGNPISVDRKHRHLVQIKCPPMLITSNTNPVTDNRWPYLNSRLMVFKFP 583

HVP39:gi|137651|sp|P24829.1| LDGYAISLDRKYKSLLQMKCPPLLITSNTNPVEDDRWPYLRSRLTVFKFP 586

HPV68:gi|260169942|gb|ACX32379.1| LDGNPISLDRKHRHLIQIKCPPMLITSNTNPVEDNRWPYLHSRLTVFKFP 579

::* .:* *:: *:*: ***:::*:* :. * **..*: *: *

HPV51:gi|137655|sp|P26544.1| NTFPFDNNGNAVYTLNDENWKNFFSTTWSRLDLEE-EEDKEN-GDPMPPF 623

HPV89:gi|6970430|dbj|BAA90737.1| NAFPFDPHGNPVYALNDVNWKNFFSTTWSRLDLEE-EEDKEN-GDPMSSF 631

HPV69:gi|6970421|dbj|BAA90729.1| NTFPFDSNGNPVYELTDVNWKSFFSTTWSRLDLEE-DADKEN-GEPLPAF 623

HPV56:tr|A9XCP9|A9XCS0 NPFPLDNNGNPVYELSNVNWKCFFTRTWSRLNLDN-DEDKENNGDAFPTF 625

HPV73:gi|1491695|emb|CAA63884.1| NPFPFDSNGNPLYQLTNENWKAFFTKTWSKLDLTE-DDDKENDGDTVQTF 639

HPV31:gi|137648|sp|P17382.1| NPFPFDKNGNPVYELSDKNWKSFFSRTWCRLNLHE-EEDKENDGDSFSTF 618

HPV35:gi|337238518|gb|AEI61432.1| NEFPFDKNGNPVYGLNDKNWKSFFSRTWCRLNLHE-EEDKENDGDAFPAF 624

HPV16:gi|242347746|gb|ACS92694.1| NEFPFDENGNPVYELNDKNWKSFFSRTWSRLSLHE-DEDKENDGDSLPTF 638

HPV33:gi|218931425|gb|ACL12328.1| NPFPFDENGNPVYAINGENWKSFFSRTWCKLDLIE-EEDKENHGGNISTF 631

HPV58:gi|260169933|gb|ACX32371.1| NPFPFDANGNPVYKINDENWKSFFSRTWCKLGLIE-EEDKENDGGNISTF 631

HPV52:gi|260169919|gb|ACX32359.1| NPFPFDENGNPIYEINNENWKSFFSRTWCKLDLIQ-EEDKENDGVDTGTF 634

HPV18:gi|60978|emb|CAA28666.1| NAFPFDKNGNPVYEINDKNWKCFFERTWSRLDLHEEEEDADTEGNPFGTF 646

HPV45:gi|145968373|gb|ABP99898.1| HAFPFDKNGNPVYEINDKNWKCFFERTWSRLDLHEDDEDADTEGNPFGTF 632

HPV59:gi|218931434|gb|ACL12336.1| NKLPFDKNRNPVYTINDRNWKCFFERTWCRLDLNEEEEDADSDGHPFAAF 633

HVP39:gi|137651|sp|P24829.1| NAFPFDQNRNPVYTINDKNWKCFFEKTWCRLDLQQDEDEGDNDENTFTTF 636

HPV68:gi|260169942|gb|ACX32379.1| NAFPFDQNRNPVYTINDKNWKCFFEKTWCKLDLQQDEDEGDNDENTFPTF 629

: :*:* : *.:* :.. *** ** **.:*.* : : : :. .*

HPV51:gi|137655|sp|P26544.1| KCVPGENTRLL-- 634

HPV89:gi|6970430|dbj|BAA90737.1| KCVPGENTRLL-- 642

HPV69:gi|6970421|dbj|BAA90729.1| KCVPGENTRLL-- 634

HPV56:tr|A9XCP9|A9XCS0 KCVPEQNTRLF-- 636

HPV73:gi|1491695|emb|CAA63884.1| KCVSGRNPRTV-- 650

HPV31:gi|137648|sp|P17382.1| KCVSGQNIRTL-- 629

HPV35:gi|337238518|gb|AEI61432.1| KCVSGQNTRTLRD 637

HPV16:gi|242347746|gb|ACS92694.1| KCVSGQNTNTL-- 649

HPV33:gi|218931425|gb|ACL12328.1| KCSAGENTRSLRS 644

HPV58:gi|260169933|gb|ACX32371.1| KCSAGQNPRHIRS 644

HPV52:gi|260169919|gb|ACX32359.1| KCSAGKNTRSIRS 647

HPV18:gi|60978|emb|CAA28666.1| KLRAGQNHRPL-- 657

HPV45:gi|145968373|gb|ABP99898.1| KCVTGQNTRPL-- 643

HPV59:gi|218931434|gb|ACL12336.1| KCVTGSNIRTL-- 644

HVP39:gi|137651|sp|P24829.1| KCVTGQNTRIL-- 647

HPV68:gi|260169942|gb|ACX32379.1| KCVTGENIRTL-- 640

* . * . .

**Multiple sequence alignment of E2 Protein dataset from all high-risk HPV strain:**

HPV31:gi|337238075|gb|AEI61089.1| ------METLSQRLNVCQDKILEHYENDSKRLCDHIDYWKHIRLECVLMY 44

HPV35:gi|337238519|gb|AEI61433.1| -----MMETLSQRLSVCQDKILEHYETDSTCLSDHIQYWKLIRLECAVFY 45

HPV16:gi|325278539|gb|ADZ04865.1| ------METLCQRFNVCQDKILTHYENDSTDLRDHIDYWKQMRLECAIYY 44

HPV33:gi|337238283|gb|AEI61249.1| ------MEEISARLNAVQEKILDLYEADKTDLPSQIEHWKLIRMECALFY 44

HPV58:gi|260169934|gb|ACX32372.1| ------MEEISARLSAVQDKILDIYEADKNDLTSQIEHWKLIRMECAIMY 44

HPV52:gi|260169920|gb|ACX32360.1| ------MESIPARLNAVQEKILDLYEADSNDLNAQIEHWKLTRMECVLFY 44

HPV18:gi|60979|emb|CAA28667.1| --MQTPKETLSERLSCVQDKIIDHYENDSKDIDSQIQYWQLIRWENAIFF 48

HPV45:gi|145968374|gb|ABP99899.1| MKMQTPKETLSERLSALQDKILDHYENDSKDINSQISYWQLIRVENAILF 50

HPV59:gi|62867023|gb|AAY17401.1| --MQTVMDTLSQRLSVLQDQILEHYENDSKDINEHINYWKLVRMENVILF 48

HPV39:gi|137679|sp|P24830.1| -MKETMMKTLSQRLNVLQDKILEYYEQDSKSIYDQINYWKCVRMENAIFY 49

HPV68:gi|260169943|gb|ACX32380.1| -MKETMMKTLSQRLNALQEKILEHYEQDSKCIKDHIKYWNSVRLENAIYY 49

HPV51:gi|137683|sp|P26547.1| ------METLCHRLNVCQEKILDCYELDSDKLVDQINYWTLLRYEAAMFY 44

HPV82:gi|6970431|dbj|BAA90738.1| ------METLCHRLNVCQEKILDCYELDSDKLVDQINYWTLVRYECAMFY 44

HPV69:gi|6970422|dbj|BAA90730.1| ------MENLCQRLNACQEKILDYYELDSDKLSDQIDYWKLVRYECAIFY 44

HPV56:tr|A9XCR4|A9XCR4 ------METLSQRLNACQNKILDCFEKDSRCIADHIEYWKAVRHENVLYY 44

HPV73:gi|1491696|emb|CAA63885.1| -----MMETLCKRLSACQDAILELYERDSVHLSDHIDHWKHVRHENVLLH 45

. : *:. *: *: :* *. : :*.:* * * .: .

HPV31:gi|337238075|gb|AEI61089.1| KAREMGIHSINHQVVPALSVSKAKALQAIELQMMLETLNNTEYKNEDWTM 94

HPV35:gi|337238519|gb|AEI61433.1| KAREMGIKTLNHQVVPTQAISKAKAMQAIELQLMLETLNTTEYRTETWTL 95

HPV16:gi|325278539|gb|ADZ04865.1| KAREMGFKHINHQVVPTLAVSKNKALQAIELQLTLKTICNSQYSNEKWTL 94

HPV33:gi|337238283|gb|AEI61249.1| TAKQMGFSHLCHQVVPSLLASKTKAFQVIELQMALETLSKSQYSASQWTL 94

HPV58:gi|260169934|gb|ACX32372.1| TARQMGISHLCHQVVPSLVASKTKAFQVIELQMALETLNASPYKTDEWTL 94

HPV52:gi|260169920|gb|ACX32360.1| KAKELGITHIGHQVVPPMAVSKAKACQAIELQLALEALNKTQYSTDGWTL 94

HPV18:gi|60979|emb|CAA28667.1| AAREHGIQTLNHQVVPAYNISKSKAHKAIELQMALQGLAQSRYKTEDWTL 98

HPV45:gi|145968374|gb|ABP99899.1| TAREHGITKLNHQVVPPSNISKSKAHKAIELQMALKGLAQSKYNNEEWTL 100

HPV59:gi|62867023|gb|AAY17401.1| AARENNIHTLNHQVVPTFLVSKNKACEAIELQMALESLAQTEFKNEQWTM 98

HPV39:gi|137679|sp|P24830.1| AARERGMHTIDHQVVPTINISKCKAYQAIELQMALESVAQTEYNTEEWTL 99

HPV68:gi|260169943|gb|ACX32380.1| AARERGMHNIDHQVVPPVNISKTKAYQAIELQMALESIAQTAYSAEEWTL 99

HPV51:gi|137683|sp|P26547.1| AARERNLRTINHQVVPATTVSKQKACQAIEMHMALQSLNKSDYNMEPWTM 94

HPV82:gi|6970431|dbj|BAA90738.1| TARERNMQTLNHQVVPASAVSKQKACQAIEMHMALESLNKSEYNMEPWTM 94

HPV69:gi|6970422|dbj|BAA90730.1| KAREGNMQCINHQVVPSTTVCKEKAWQAIELHIALQSLMNSEYGKEKWTM 94

HPV56:tr|A9XCR4|A9XCR4 KARENDITVLNHQMVPCLQVCKAKACSAIEVQIALESLSTTIYNNEEWTL 94

HPV73:gi|1491696|emb|CAA63885.1| KAREMGLQTVNNQAVPSLAVSRSKGYNAIEMQIALESLNESLYNTEEWTL 95

*:: .: : :* ** .: *. ..**::: *: : : : . **:

HPV31:gi|337238075|gb|AEI61089.1| QQTSLELYLTAPTGCLKKHGYTVQVQFDGDVHNTMHYTNWKFIYLCIDG- 143

HPV35:gi|337238519|gb|AEI61433.1| QETSIELYTTVPQGCFKKHGVTVEVQFDGDKQNTMHYTNWTHIYLLEDS- 144

HPV16:gi|325278539|gb|ADZ04865.1| QDVSLEVYLTAPTGCIKKHGYTVEVQFDGDICNTMHYTNWKYIYICEET- 143

HPV33:gi|337238283|gb|AEI61249.1| QQTSLEVWLCEPPKCFKKQGETVTVQYDNDKKNTMDYTNWGEIYIIEED- 143

HPV58:gi|260169934|gb|ACX32372.1| QQTSLEVWLSEPQKCFKKKGITVTVQYDNDKANTMDYTNWSEIYIIEET- 143

HPV52:gi|260169920|gb|ACX32360.1| QQTSLEMWRAEPQKYFKKHGYTITVQYDNDKNNTMDYTNWKEIYLLGEC- 143

HPV18:gi|60979|emb|CAA28667.1| QDTCEELWNTEPTHCFKKGGQTVQVYFDGNKDNCMTYVAWDSVYYMTDAG 148

HPV45:gi|145968374|gb|ABP99899.1| QDTCEELWNTEPSQCFKKGGKTVHVYFDGNKDNCMNYVVWDSIYYISETG 150

HPV59:gi|62867023|gb|AAY17401.1| QETCQELWQTAPKKCFKKQGITVEVRFDCSKENTMHYTSWKFIYYVNDVG 148

HPV39:gi|137679|sp|P24830.1| KDTSNELWHTQPKQCFKKQGTTVEVWYDGDKCNAMNYVLWGAIYYKNNID 149

HPV68:gi|260169943|gb|ACX32380.1| RDTSNELWHTKPKQCFKKHGVTVEVWYDGDKSNSMHYVVWGTIYFKNSTD 149

HPV51:gi|137683|sp|P26547.1| RETCYELWCVAPKQCFKKGGITVTVIFDGNKDNAMDYTSWKFIYIYDND- 143

HPV82:gi|6970431|dbj|BAA90738.1| RDTCYELWGEAPKHCFKKGGKTVTVMFDGNKDNTMDYTCWTYVYIYKED- 143

HPV69:gi|6970422|dbj|BAA90730.1| RDTCYELYVTEPKHCFKKEGTTVTVVFDCNKENTMDYIRWTSVYYKTDM- 143

HPV56:tr|A9XCR4|A9XCR4 RDTCEELWLTEPKKCFKKEGQHIEVWFDGSKNNCMQYVAWKYIYYNGDC- 143

HPV73:gi|1491696|emb|CAA63885.1| QHTSWELWVTEPKQCFKKDGKTVEVRYDCEKDNSMQYVFWTHIYCWYEG- 144

:... *:: * :** * : * :* . * * * * :* .

HPV31:gi|337238075|gb|AEI61089.1| QCTVVEGQVNCKGIYYVHEG-HITYFVNFTEEAKKYGTGKKWEVHAGGQV 192

HPV35:gi|337238519|gb|AEI61433.1| TCTVVKGLVNYKGIYYVHQG-VETYYVNFREEAKKYGKKNIWEVHVGGQV 193

HPV16:gi|325278539|gb|ADZ04865.1| SVTVVEGQVDYYGLYYVHEG-IQTYFVQFKDDAEKYSKNKVWEVHAGGQV 192

HPV33:gi|337238283|gb|AEI61249.1| TCTMVTGEVDYIGMYYVHNC-EKVYFKYFKEDAAKYSKTQMWEVHVGGQV 192

HPV58:gi|260169934|gb|ACX32372.1| TCTLVAGEVDYVGLYYIHGN-EKTYFKYFKEDAKKYSKTQLWEVHVGSRV 192

HPV52:gi|260169920|gb|ACX32360.1| ECTIVEGQVDYYGLYYWCDG-EKTYFVKFSNDAKQYCATGVWEVHVGGRV 192

HPV18:gi|60979|emb|CAA28667.1| TWDKTATCVSHRGLYYVKEG-YNTFYIEFKSECEKYGNTGTWEVHFGNNV 197

HPV45:gi|145968374|gb|ABP99899.1| IWEKTAACVSYWGVYYIKDG-NTTYYVQFKSECEKYGNSNTWEVQYGGNV 199

HPV59:gi|62867023|gb|AAY17401.1| QWCKTTGSVDFWGLYYNVEE-EQVYYVKFIHDAKKYGTTDKWEVHFNGKV 197

HPV39:gi|137679|sp|P24830.1| IWCKTEGCVDYWGIYYMNEH-LKVYYEVFIQDAERYGTSGKWEVHYNGNI 198

HPV68:gi|260169943|gb|ACX32380.1| TWCKTKGCVDYWGVYYMYEK-QKTYYERFMNDAQLYGTSGKWDVHYNGNI 198

HPV51:gi|137683|sp|P26547.1| KWVKTNGNVDYTGIYYTVNS-KKEYYVQFKDEAKIYG-AQQWEVYMYGTV 191

HPV82:gi|6970431|dbj|BAA90738.1| RWVKTQGNVDYTGIYYKPDV-NKEYYVTFKDEAKKYG-AQQWEVYMCGNV 191

HPV69:gi|6970422|dbj|BAA90730.1| GWCKTNGEVDYKGIYYMHGC-CKQYYVDFKQEALKYGTGLQWDVHVCGQV 192

HPV56:tr|A9XCR4|A9XCR4 GWQKVCSGVDYRGIYYVHDG-HKTYYTDFEQEAKKFGCKNIWEVHMENES 192

HPV73:gi|1491696|emb|CAA63885.1| GWAKVGSKIDYNGIYYETDDEEKVYYTRFDTDAKRYGVKGIWEVHMGGQV 194

. :. *:** :: * :. : *:* .

HPV31:gi|337238075|gb|AEI61089.1| IVFPESVFSS--DEISFAGIVTKLPTANNTTTSNSKTCALGTSEGVRRAT 240

HPV35:gi|337238519|gb|AEI61433.1| IVCPESVFSS--TELSTAEIATQL-HAYNTTETHTKACSVGTTETQK--- 237

HPV16:gi|325278539|gb|ADZ04865.1| ILCPTSVFSS--DEVSSAEIIRQH-LANHSAATHTKTVALGTKETQT--- 236

HPV33:gi|337238283|gb|AEI61249.1| IVCPTSISS---NQISTTETADIQ----TDNANRPPQAAA---------- 225

HPV58:gi|260169934|gb|ACX32372.1| IVCPTSIPS---DQISTTETADPK----TTEATNNESTQG---------- 225

HPV52:gi|260169920|gb|ACX32360.1| IVCPASVSS---NEVSTTETAVHL----CTETSKTSALSVGAKDTHLQP- 234

HPV18:gi|60979|emb|CAA28667.1| IDCNDSMCSTSDDTVSATQLVKQL---QHTPSPYSSTVSVGTAKTYGQ-T 243

HPV45:gi|145968374|gb|ABP99899.1| IDCNDSMCSTSDDTVSATQIVRQL---QHASTSTPKTASVGTPKPHIQ-T 245

HPV59:gi|62867023|gb|AAY17401.1| IDCYDSMCSTSDEQVSTSGSSEQL---SYPSATPPEATYVGPQTSNCP-T 243

HPV39:gi|137679|sp|P24830.1| IHCPDSMCSTSDGSVPTTELTTEL---SNTTATHSTATTPCTQKTIP--P 243

HPV68:gi|260169943|gb|ACX32380.1| IHCPDSMCSTTDGTVSTSESIAEL---QNTTATHTTAGAPCTKKTHS--T 243

HPV51:gi|137683|sp|P26547.1| ITCPEYVSSTCSDALSTTTTVEQL---SNTPTTNPLTTCVG--AKEAQ-T 235

HPV82:gi|6970431|dbj|BAA90738.1| ITCPEYVSSTYSTPSPSTTTVEQL---SNTPTTNTYSASAG--TTEAQ-T 235

HPV69:gi|6970422|dbj|BAA90730.1| ICCPEFVSSTSGDQISTAQTAEPL---SNNTTQTPETTCVPRCTEETP-A 238

HPV56:tr|A9XCR4|A9XCR4 IYCPDSVSSTCRYNVSPVETVNEY--NTHKTTTTTSTSVGNQDAAVSHRP 240

HPV73:gi|1491696|emb|CAA63885.1| ICCAP-VSSACEVSIP--EIVNP----LHTTTTNTTTTCTNVDTGVPSR- 236

* : * .

HPV31:gi|337238075|gb|AEI61089.1| TSTKRPRTEPEHRNTHHPNKLLRGD-SVDSVNCG--VISAAACTNQTRAV 287

HPV35:gi|337238519|gb|AEI61433.1| TNHKRPRGGTELP--CNPTKRVRLS-AVDSVDRG--VYSTSDCTNKDRCG 282

HPV16:gi|325278539|gb|ADZ04865.1| -TIQRPRSEPDTGNPCHTNKLLHRD-SVDSAP----ILTAVNSSHKGRIN 280

HPV33:gi|337238283|gb|AEI61249.1| -KRRRSADTTDTG--QPLTKLFCADPALDNRTAR----TATNCTNKQRTV 268

HPV58:gi|260169934|gb|ACX32372.1| -TKRRRLDLPDSRD-NTQYSTKYTDCAVDSRPRGGGLHSTTNCTYKGRNV 273

HPV52:gi|260169920|gb|ACX32360.1| PQKRRRPDVTDSRNTKYPNNLLRGQQSVDSTTRG--LVTATECTNKGRVA 282

HPV18:gi|60979|emb|CAA28667.1| SAATRPGHCGLAEKQHCGP------VNPLLG------AATPTGNNKRRKL 281

HPV45:gi|145968374|gb|ABP99899.1| PATKRPRQCGLTEQHH-GL------VNTHVHN-P--LLCSSTSNNKRRKV 285

HPV59:gi|62867023|gb|AAY17401.1| KTGKRPRQCGYTQHPQSTS------VSVDHCDNP--VVRLHPGNNPRRHI 285

HPV39:gi|137679|sp|P24830.1| PSRKRPRQCAVTEPTEPDG------VSLDHLNNP--LHSNSTGHNTRRYL 285

HPV68:gi|260169943|gb|ACX32380.1| SSWKHPRQYGITEPSEPND------VSVDGVNLP--LLSRSAGHNKRRNL 285

HPV51:gi|137683|sp|P26547.1| -QQRKRQRLTEPDSSTISP------LSVDNTNNQ--IHCGSGSTNTGGHQ 276

HPV82:gi|6970431|dbj|BAA90738.1| PQQRKRQRLAEPDSSTVTP------LSVDTCDHQ--IHCGTAGPNTGGHL 277

HPV69:gi|6970422|dbj|BAA90730.1| PETAKRRRLSGPNTPVTTTSVTRPRSSLEHTDNV--LHSNSGSNNPGRGT 286

HPV56:tr|A9XCR4|A9XCR4 GKRPRLRESEFDSSRESHAK----CVTTHTHISDTDNTDSRSRSINNNNH 286

HPV73:gi|1491696|emb|CAA63885.1| -KRQRQCDS-----DQRPLD----CLHNLHPT-----TESCTQCTTHN-- 269

:

HPV31:gi|337238075|gb|AEI61089.1| SCPATTPIIHLKGDANILKCLRYRLSK-YKQLYEQVSSTWHWTC-TDGKH 335

HPV35:gi|337238519|gb|AEI61433.1| SCSTTTPIVHLKGDANTLKCLRYRLGK-YKALYQDASSTWRWTC-TNDKK 330

HPV16:gi|325278539|gb|ADZ04865.1| CNSNTTPIVHLKGDANTLKCLRYRFKK-HCKLYTAVSSTWHWTG-HNVKH 328

HPV33:gi|337238283|gb|AEI61249.1| CSSNVAPIVHLKGESNSLKCLRYRLKP-YKELYSCMSSTWHWTS-DNKNS 316

HPV58:gi|260169934|gb|ACX32372.1| CSSKVAPILHLKGEPNSLKWFRYRLKP-FKDLYCNISSTWHWTS-DDKGD 321

HPV52:gi|260169920|gb|ACX32360.1| HTTCTAPIIHLKGDPNSLKCLRYRVKP-HNSLYVQISSTWHWTSNECTNT 331

HPV18:gi|60979|emb|CAA28667.1| CSGNTTPIIHLKGDRNSLKCLRYRLRK-HSDHYRDISSTWHWTG-AGNEK 329

HPV45:gi|145968374|gb|ABP99899.1| CSGNTTPIIHLKGDKNSLKCLRYRLRK-YADHYSEISSTWHWTG-CKKN- 332

HPV59:gi|62867023|gb|AAY17401.1| PCSNTTPIIHLKGDKNGLKCLRYRLKK-VQWLFENISSTWHWTGNRGSAK 334

HPV39:gi|137679|sp|P24830.1| SCGNTTPIIHLKGDKNGLKCLRYRLQK-YDTLFENISCTWHWIRGKGTKN 334

HPV68:gi|260169943|gb|ACX32380.1| CCGDTTPIVHLKGDKNGLKCFRYRLQK-YSALYENISCTWHWIRGRGSTN 334

HPV51:gi|137683|sp|P26547.1| SATQTAFIVHLKGDTNCLKCFRYRFTK-HKGLYKNVSSTWHWTS---NTK 322

HPV82:gi|6970431|dbj|BAA90738.1| SATKTAFIVHLKGATNCLKCLRYRFAK-HRNLFKEVSSTWHWTS---NTK 323

HPV69:gi|6970422|dbj|BAA90730.1| CSDQTVFIVHLKGDTNCLKCLRYRFKK-HKGLYCKVSSTWHWTS---NTN 332

HPV56:tr|A9XCR4|A9XCR4 PGDKTTPVVHLKGEPNRLKCCRYRFQK-YKTLFVDVTSTYHWTS--TDNK 333

HPV73:gi|1491696|emb|CAA63885.1| ----VAPIVHLKGDKNSLKCFRYRLHKGYSHLFKNVTTTWHWTN--TTNS 313

.. ::**** * ** ***. : : *::*

HPV31:gi|337238075|gb|AEI61089.1| KNAIVTLTYISTSQRDDFLNTVKIPNTVSVSTGYMTI 372

HPV35:gi|337238519|gb|AEI61433.1| QIAIVTLTYTTEYQRDKFLTTVKIPNTVTVSKGYMSI 367

HPV16:gi|325278539|gb|ADZ04865.1| KSAIVTLTYDSECQREQFLSQVKIPKTITVSTGFMSI 365

HPV33:gi|337238283|gb|AEI61249.1| KNGIVTVTFATEQQQQMFLGTVKIPPTVQISTGFMTL 353

HPV58:gi|260169934|gb|ACX32372.1| KVGIVTVTYTTETQRQLFLNTVKIPPTVQISTGVMSL 358

HPV52:gi|260169920|gb|ACX32360.1| KLGIVTITYSDEAQREQFLKTVKIPNTVQVIQGVMSL 368

HPV18:gi|60979|emb|CAA28667.1| -TGILTVTYHSETQRTKFLNTVAIPDSVQILVGYMTM 365

HPV45:gi|145968374|gb|ABP99899.1| -TGILTVTYNSEVQRNTFLDVVTIPNSVQISVGYMTM 368

HPV59:gi|62867023|gb|AAY17401.1| -TGILTLTYTSETQRNEFLDTVKIPNSVQIQVGYMSV 370

HPV39:gi|137679|sp|P24830.1| -AGILTVTYATESQRQKFLDTVKIPSSVHVSLGYMTL 370

HPV68:gi|260169943|gb|ACX32380.1| -TGILTVTYVSESQRQKFLETVKIPSSVTVSLGYMTL 370

HPV51:gi|137683|sp|P26547.1| -TGIVTIVFDSAHQRETFIKTIKVPPSVTLSLGIMTL 358

HPV82:gi|6970431|dbj|BAA90738.1| -AGIVTITFDSAHQRQKFIDTVKVPSSVTVSLGIMTV 359

HPV69:gi|6970422|dbj|BAA90730.1| -QGIVTITFDSETQRASFLTTVKIPQSITSTLGIMSL 368

HPV56:tr|A9XCR4|A9XCR4 NYSIITIIYKDETQRNSFLSHVKIPVVYRLVWDK--- 367

HPV73:gi|1491696|emb|CAA63885.1| KCGVITLMFTTVLQQQHFLQHVKIPQTIVVTSGYMSL 350

.::*: : *: *: : :* .

**Multiple sequence alignment of E6 Protein dataset from all high-risk HPV strain:**

HPV16:gi|325070944|gb|ADY75575.1| MHQKRTAMFQDPQERPRKLPQLCTELQTTIHDIILECVYCKQQLLRREVY 50

HPV35:gi|66933414|gb|AAY58345.1|| -------MFQDPAERPYKLHDLCNEVEESIHEICLNCVYCKQELQRSEVY 43

HPV33:gi|256260301|gb|ACU65221.1| -------MFQDTEEKPRTLHDLCQALETTIHNIELQCVECKKTLQRSEVY 43

HPV58:gi|66933386|gb|AAY58331.1| -------MFQDAEEKPRTLHDLCQALETSVHEIELKCVQCKKTLQRSEVY 43

HPV52:gi|66933374|gb|AAY58325.1| -------MFEDPATRPRTLHELCEVLEESVHEIRLQCVQCKKELQRREVY 43

HPV31:gi|66933402|gb|AAY58339.1| -------MFKNPAERPRKLHELSSALEIPYDELRLNCVYCKGQLTETEVL 43

HPV73:gi|1491693|emb|CAA63882.1| ------MLFPNSEERPYKLQALCDEVNISIHDINLDCVFCQRGLYRSEVY 44

HPV51:gi|137774|sp|P26554.1| -------MFEDKRERPRTLHELCEALNVSMHNIQVVCVYCKKELCRADVY 43

HPV82:gi|6970428|dbj|BAA90735.1| -------MFEDIRERPRTLHELCEACNTSMHNIQVLCVYCKKELCRADVY 43

HPV69:gi|6970419|dbj|BAA90727.1| -------MFQDPRERPRTIHELCEALNTPLQSLQVQCVYCKKTLEWADVY 43

HPV56:gi|336390152|gb|AEI54137.1| ----MEPQFNNPQERPRSLHHLSEVLEIPLIDLRLSCVYCKKELTRAEVY 46

HPV18:gi|5748504|emb|CAB53096.1| -----MARFEDPTRRPYKLPDLCTELNTSLQDIEITCVYCKTVLELTEVF 45

HPV45:gi|145968371|gb|ABP99896.1| -----MARFDDPTQRPYKLPDLCTELNTSLQDVSIACVYCKATLERTEVY 45

HPV39:gi|137767|sp|P24835.1| -----MARFHNPAERPYKLPDLCTTLDTTLQDITIACVYCRRPLQQTEVY 45

HPV68:gi|3005942|emb|CAA74931.1| -----MALFHNPEERPYKLPDLCRTLDTTLHDVTIDCVYCRRQLQRTEVY 45

HPV59:gi|218931432|gb|ACL12334.1| -----MARFEDPTQRPYKLPDLSTTLNIPLHDIRINCVFCKEELQEREVF 45

* : :* .: *. : . .: : ** *: * :*

HPV16:gi|325070944|gb|ADY75575.1| DFAFRDLCIVYRDGNPYAVCDKCLKFYSKISEYRHYCYSLYGTTLEQQYN 100

HPV35:gi|66933414|gb|AAY58345.1|| DFACYDLCIVYREGQPYGVCMKCLKFYSKISEYRWYRYSVYGETLEKQCN 93

HPV33:gi|256260301|gb|ACU65221.1| DFAFADLTVVYREGNPFGICKLCLRFLSKLSEYRHYNYSLYGNTLEQTVN 93

HPV58:gi|66933386|gb|AAY58331.1| DFVFADLRIVYRDGNPFAVCKVCLRLLSKISEYRHYNYSLYGETLEQTLK 93

HPV52:gi|66933374|gb|AAY58325.1| KFLFTDLRIVYRDNNPYGVCIMCLRFLSKISEYRHYQYSLYGKTLEERVR 93

HPV31:gi|66933402|gb|AAY58339.1| DFAFTDLTIVYRDDTPYGVCTKCLRFYSKVSEFRWYRYSVYGTTLEKLTN 93

HPV73:gi|1491693|emb|CAA63882.1| DFAFSDLCIVYRKDKPYGVCQPCLKFYSKIREYRRYRQSVYGTTLENLTN 94

HPV51:gi|137774|sp|P26554.1| NVAFTEIKIVYRDNNPYAVCKQCLLFYSKIREYRRYSRSVYGTTLEAITK 93

HPV82:gi|6970428|dbj|BAA90735.1| NVAFTELRIVYRDNTPYAACKKCLMFYSRIREYRRYSRSVYGATLEAITN 93

HPV69:gi|6970419|dbj|BAA90727.1| NFAICDLRIVYRNDSAYGACKKCIIFYSKIIEYRRYTSSVYGATLEARPK 93

HPV56:gi|336390152|gb|AEI54137.1| NFACTELKLVYRDDFPYAVCRVCLLFYSKVRKYRYYDYSVYGATLESITK 96

HPV18:gi|5748504|emb|CAB53096.1| EFAFKDLFVVYRDSIPHAACHKCIDFYSRIRELRHYSDSVYGDTLEKLTN 95

HPV45:gi|145968371|gb|ABP99896.1| QFAFKDLFIVYRDCIAYAACHKCIDFYSRIRELRYYSNSVYGETLEKITN 95

HPV39:gi|137767|sp|P24835.1| EFAFSDLYVVYRDGEPLAACQSCIKFYAKIRELRYYSDSVYATTLENITN 95

HPV68:gi|3005942|emb|CAA74931.1| EFAFGDLNVVYRDGVPLAACQSCIKFYAKIRELRYYSESVYATTLETITN 95

HPV59:gi|218931432|gb|ACL12334.1| EFAFNDLFIVYRDCTPYAACLKCISFYARVRELRYYRDSVYGGTLEAETK 95

.. :: :***. . . * *: : ::: : * * *:*. *** .

HPV16:gi|325070944|gb|ADY75575.1| KPLCDLLIRCINCQKPLCPEEKQRHLDKKQRFHNIRGRWTGRCMSCCRSS 150

HPV35:gi|66933414|gb|AAY58345.1|| KQLCHLLIRCITCQKPLCPVEKQRHLEEKKRFHNIGGRWTGRCMSCWKP- 142

HPV33:gi|256260301|gb|ACU65221.1| KPLNEILIRCIICQRPLCPQEKKRHVDLNKRFHNISGRWAGRCAVCWRS- 142

HPV58:gi|66933386|gb|AAY58331.1| KCLNEILIRCIICQRPLCPQEKKRHVDLNKRFHNISGRWTGRCAVCWRP- 142

HPV52:gi|66933374|gb|AAY58325.1| KPLSEITIRCIICQTPLCPEEKERHVNANKRFHNIMGRWTGRCSECWRP- 142

HPV31:gi|66933402|gb|AAY58339.1| KGICDLLIRCITCQRPLCPEEKQRHLDKKKRFHNIGGRWTGRCIVCWRR- 142

HPV73:gi|1491693|emb|CAA63882.1| KQLCNILIRCGKCQKPLCPLEKQKHVDEKKRFHQIAEQWTGRCTRCWRP- 143

HPV51:gi|137774|sp|P26554.1| KSLYDLSIRCHRCQRPLGPEEKQKLVDEKKRFHEIAGRWTGQCANCWQRT 143

HPV82:gi|6970428|dbj|BAA90735.1| KSLYELLIRCHRCQRPLGPEEKQKVVDDKKRFHEIAGRWTGQCANCRKPP 143

HPV69:gi|6970419|dbj|BAA90727.1| RSLCNLLIRCHRCQIPLGPEEKQRIVDEKRRFHEIAGYWKGLCTNCWRPR 143

HPV56:gi|336390152|gb|AEI54137.1| KQLCDLLIRCYRCQSPLTPEEKQLHCDRKRRFHLIAHGWTGSCLGCWRQT 146

HPV18:gi|5748504|emb|CAB53096.1| TGLYNLLIRCLRCQKPLNPAEKLRHLNEKRRFHKIAGHYRGQCHSCCNRA 145

HPV45:gi|145968371|gb|ABP99896.1| TELYNLLIRCLRCQKPLNPAEKRRHLKDKRRFHSIAGQYRGQCNTCCDQA 145

HPV39:gi|137767|sp|P24835.1| TKLYNLLIRCMCCLKPLCPAEKLRHLNSKRRFHKIAGSYTGQCRRCWTTK 145

HPV68:gi|3005942|emb|CAA74931.1| TKLYDLSIRCMCCLKPLSPAEKLRHLNSKRRFHKIAGNFTGQCRHCWTSK 145

HPV59:gi|218931432|gb|ACL12334.1| TPLHELLIRCYRCLKPLCPTDKLKHITEKRRFHNIAGIYTGQCRGCRTRA 145

: .: *** * ** * :* ::*** * : * * *

HPV16:gi|325070944|gb|ADY75575.1| -------RTRRETQL 158

HPV35:gi|66933414|gb|AAY58345.1|| --------TRRETEV 149

HPV33:gi|256260301|gb|ACU65221.1| --------RRRETAL 149

HPV58:gi|66933386|gb|AAY58331.1| --------RRRQTQV 149

HPV52:gi|66933374|gb|AAY58325.1| --------RP-VTQV 148

HPV31:gi|66933402|gb|AAY58339.1| --------PRTETQV 149

HPV73:gi|1491693|emb|CAA63882.1| ----------SATVV 148

HPV51:gi|137774|sp|P26554.1| -------RQRNETQV 151

HPV82:gi|6970428|dbj|BAA90735.1| -------RQRSETQV 151

HPV69:gi|6970419|dbj|BAA90727.1| -------REATETQV 151

HPV56:gi|336390152|gb|AEI54137.1| S------REPRESTV 155

HPV18:gi|5748504|emb|CAB53096.1| RQERLQR--RRETQV 158

HPV45:gi|145968371|gb|ABP99896.1| RQERLRR--RRETQV 158

HPV39:gi|137767|sp|P24835.1| REDRRLT--RRETQV 158

HPV68:gi|3005942|emb|CAA74931.1| REDRRRT--RQETQV 158

HPV59:gi|218931432|gb|ACL12334.1| RHLRQQRQARSETLV 160

: :

**Multiple sequence alignment of E7 Protein dataset from all high-risk HPV strain:**

HPV18:gi|137792|sp|P06788.2 MHGPKATLQDIVLHLEPQNEI-PVDLLCHEQLS----DSEEEN--DEIDG 43

HPV45:gi|549287|sp|P21736.2 MHGPRETLQEIVLHLEPQNELDPVDLLCYEQLS----ESEEEN--DEADG 44

HPV59:HPV59:tr|Q81965| MHGPKATLCDIVLDLEPQNYE-EVDLVCYEQLP----DSDSENEKDEPDG 45

HVP39:gi|137798|sp|P24837.1 MRGPKPTLQEIVLDLCPYNEIQPVDLVCHEQLG----ESEDEID-EPDHA 45

HPV68:gi|1718147|sp|P54668.1 MHGPKPTVQEIVLELCPYNEIQPVDLVCHEQLG----DSDDEID-EPDHA 45

HPV31:gi|137795|sp|P17387.1 MRGETPTLQDYVLDLQP----EATDLHCYEQLP----DSSDEE--DVIDS 40

HPV35:gi|137797|sp|P27230.1 MHGEITTLQDYVLDLEP----EATDLYCYEQLC----DSSEEEE-DTIDG 41

HPV16:gi|137791|sp|P03129.1 MHGDTPTLHEYMLDLQP----ETTDLYCYEQLN----DSSEEE--DEIDG 40

HPV33:gi|137796|sp|P06429.1 MRGHKPTLKEYVLDLYP----EPTDLYCYEQLS----DSSDEDE--GLDR 40

HPV58:gi|137805|sp|P26557.1 MRGNNPTLREYILDLHP----EPTDLFCYEQLC----DSSDEDEI-GLDG 41

HPV52:gi|549289|sp|P36831.1 MRGDKATIKDYILDLQP----ETTDLHCYEQLG----DSSDEEDTDGVDR 42

HPV73:HPV73:tr|Q82006| MHGKKTTLQDITLDLKP---TTEIDLTCYESLD----NSEDED---ETDS 40

HPV51:gi|137803|sp|P26558.1 MRGNVPQLKDVVLHLTPQ---TEIDLQCYEQFD----SSEEEDEVDNMRD 43

HPV82:HPV82:tr|Q993Z9| MRGNVPQVKDIVLELTPQ---PEIDLQCYEQFD----SSDEEDEVDNMRD 43

HPV69:gi|76363469|sp|Q9JH50.1 MHGDTINIQDVILDLVPQ---PEIDLQCYEQLDYEQFDSSEEDETDNVRN 47

HPV56:gi|549291|sp|P36833.1 MHGKVPTLQDVVLELTPQ---TEIDLQCNEQLDSS--EDEDEDEVDHLQE 45

*:* : : *.* * ** * *.: ....*

HPV18:gi|137792|sp|P06788.2 VN--HQHLPARRAEPQRHTMLCMCCKCEARIKLVVESSADDLRAFQQLFL 91

HPV45:gi|549287|sp|P21736.2 VS--HAQLPARRAEPQRHKILCVCCKCDGRIELTVESSAEDLRTLQQLFL 92

HPV59:tr|Q81965| VN--HPLLLARRAEPQRHNIVCVCCKCNNQLQLVVETSQDGLRALQQLFM 93

HVP39:gi|137798|sp|P24837.1 VN-HQHQLLARRDEPQRHTIQCSCCKCNNTLQLVVEASRDTLRQLQQLFM 94

HPV68:gi|1718147|sp|P54668.1 VNHHQHLLLARRDEQQRHRIQCLCCKCNKALQLVVEASRDNLRTLQQLFM 95

HPV31:gi|137795|sp|P17387.1 PA------GQAEPDTSNYNIVTFCCQCKSTLRLCVQSTQVDIRILQELLM 84

HPV35:gi|137797|sp|P27230.1 PA------GQAKPDTSNYNIVTSCCKCEATLRLCVQSTHIDIRKLEDLLM 85

HPV16:gi|137791|sp|P03129.1 PA------GQAEPDRAHYNIVTFCCKCDSTLRLCVQSTHVDIRTLEDLLM 84

HPV33:gi|137796|sp|P06429.1 PD------GQAQPATADYYIVTCCHTCNTTVRLCVNSTASDLRTIQQLLM 84

HPV58:gi|137805|sp|P26557.1 PD------GQAQPATANYYIVTCCYTCGTTVRLCINSTTTDVRTLQQLLM 85

HPV52:gi|549289|sp|P36831.1 PD------GQAEQATSNYYIVTYCHSCDSTLRLCIHSTATDLRTLQQMLL 86

HPV73:tr|Q82006| HL-------DRQAERECYRIVTDCTKCQCTVCLAIESNKADLRVIEELLM 83

HPV51:gi|137803|sp|P26558.1 QL-----PERRAGQATCYRIEAPCCRCSSVVQLAVESSGDTLRVVQQMLM 88

HPV82:tr|Q993Z9| Q------PARQAGQATCYRIKVQCCRCSSLLQLAVESSGDNLRIFQQLLM 87

HPV69:gi|76363469|sp|Q9JH50.1 Q------QARQAEQEACYRIEAECCVCNSIVQLAVLSSRQNVRAVEQLLM 91

HPV56:gi|549291|sp|P36833.1 RPQ----QARQAKQHTCYLIHVPCCECKFVVQLDIQSTKEDLRVVQQLLM 91

: : * * : * : :. :* .:::::

HPV18:gi|137792|sp|P06788.2 NTLSFVCPWCASQQ- 105

HPV45:gi|549287|sp|P21736.2 STLSFVCPWCATNQ- 106

HPV59:tr|Q81965| DTLSFVCPLCAANQ- 107

HVP39:gi|137798|sp|P24837.1 DSLGFVCPWCATANQ 109

HPV68:gi|1718147|sp|P54668.1 DSLNFVCPWCATETQ 110

HPV31:gi|137795|sp|P17387.1 GSFGIVCPNCSTRL- 98

HPV35:gi|137797|sp|P27230.1 GTFGIVCPGCSQRA- 99

HPV16:gi|137791|sp|P03129.1 GTLGIVCPICSQKP- 98

HPV33:gi|137796|sp|P06429.1 GTVNIVCPTCAQQ-- 97

HPV58:gi|137805|sp|P26557.1 GTCTIVCPSCAQQ-- 98

HPV52:gi|549289|sp|P36831.1 GTLQVVCPGCARL-- 99

HPV73:tr|Q82006| GTLGIVCPNCSRNL- 97

HPV51:gi|137803|sp|P26558.1 GELSLVCPCCANN-- 101

HPV82:tr|Q993Z9| GDLSLVGPCCANN-- 100

HPV69:gi|76363469|sp|Q9JH50.1 GDVSLVCHQCATY-- 104

HPV56:gi|549291|sp|P36833.1 GALTVTCPLCASSN- 105

. .. *:

The multiple alignment files are prepared using ClustalW tool.

***** indicates that amino acid residue is same for all the aligned sequences at that position.

**:** indicates that amino acid residue is conserved in all the sequences at that position.

**.** indicates that amino acid residue is semi-conserved in all the sequences at that position.
